# Machine learning-guided in Silico identification of Na⁺-NQR inhibitors from *Berberis vulgaris* and *Hydrastis Canadensis* phytochemicals against *Vibrio cholerae*

**DOI:** 10.1038/s41598-025-23546-2

**Published:** 2025-11-12

**Authors:** Leena H. Bajrai, Mai M. El-Daly, Ibrahim A. AL-Zahrani, Amira M. Alghamdi, Isra M. Alsaady, Vivek Dhar Dwivedi, Esam I. Azhar

**Affiliations:** 1 Department of Biochemistry, Faculty of Sciences, King Abdulaziz University, Jeddah, 21362 Saudi Arabia; 2 Special Infectious Agents Unit – BSL3, King Fahd Medical Research Center, King Abdulaziz University, Jeddah, 21362 Saudi Arabia; 3 Department of Medical Laboratory Sciences, Faculty of Applied Medical Sciences, King Abdulaziz University, Jeddah, 21362 Saudi Arabia; 4 Center for Global Health Research, Saveetha Institute of Medical and Technical Sciences, Saveetha Medical College and Hospitals, Saveetha University, Chennai, 605102 India; 5 Bioinformatics Research Division, Quanta Calculus, Greater Noida, 201310 India

**Keywords:** Cholera, Berberis vulgaris, Hydrastis canadensis, DFT analysis, MD simulation, Network pharmacology, Na- NQR enzyme, Drug discovery, Virtual screening

## Abstract

Cholera, a life-threatening diarrheal disease caused by Vibrio cholerae, remains a global health concern. Traditional medicinal plants such as *Berberis vulgaris* (barberry) and *Hydrastis canadensis* (goldenseal) have long been used for their antimicrobial properties. This study employed integrated computational approaches to identify potential anti-cholera compounds from these plants by targeting sodium-pumping NADH-ubiquinone oxidoreductase (Na⁺-NQR). Virtual screening identified compounds **122623**, **197835**, and **638024**, along with the control Korormicin, exhibiting favorable binding affinities and hydrogen bonding interactions. Machine learning-based prediction models were further applied to assess functional activity and binding affinity. The study incorporated ADMET (Absorption, Distribution, Metabolism, Excretion, and Toxicity) profiling and density functional theory (DFT) calculations to evaluate physicochemical properties and electronic characteristics of the compounds. Molecular dynamics (MD) simulations demonstrated stable RMSD values for the ligands, with **122623** exhibiting the lowest RMSD (0.3–0.5 nm), closely resembling the control (0.3–0.4 nm), suggesting stable binding. Principal component analysis (PCA) showed tight conformational clusters for complexes with **122623** and **197835**, while free energy landscape (FEL) analysis revealed deep energy minima, supporting complex stability. MM/GBSA calculations showed that **122623** had the lowest binding free energy (-38.71 kcal/mol), followed by **638024** (-35.14 kcal/mol) and **197835** (-30.68 kcal/mol). Per-residue energy decomposition identified key residues (Phe137, Val155, Phe159, Phe160) involved in ligand binding. Network pharmacology analysis predicted additional gene targets for the selected compounds, providing insights into their broader therapeutic relevance. Collectively, compounds **122623**, **197835**, and **638024** derived from *Hydrastis canadensis* and *Berberis vulgaris* demonstrated promising inhibitory interactions with the Na⁺-NQR enzyme, suggesting their potential as anti-cholera agents.

**Supplementary Information:**

The online version contains supplementary material available at 10.1038/s41598-025-23546-2.

## Introduction

Cholera, a highly severe gastrointestinal disease, is spread through the assimilation of food or water polluted with *Vibrio cholerae*, a pathogenic bacterium. The previously stated studies mentioned that this disease problem affects public health, exhibiting not only a medical emergency but also exemplifying deeper concerns linked to socioeconomic injustice and insufficient progress. Moreover, current epidemiological evidence proposes a significant increase in the global occurrence of cholera. The annual morbidity rate of cholera is currently estimated to range between 1.3 and 4.0 million cases, and it is responsible for a mortality rate that ranges from 21,000 to 143,000 deaths each year^1^. When compared to the total number of cases of cholera reported in 2021, the World Health Organization (WHO) reports that the total cholera cases reported in 2022 more than doubled. This increase was observed in 44 countries, and when compared to the 35 countries that reported cases in the previous year, this is a 25% increase^2^. According to the most recent report from the World Health Organization (WHO), cholera remains a serious global health concern. As of 24 January 2025, a cumulative total of 804,721 suspected cholera cases and 5,805 deaths have been reported from 33 countries across five WHO regions during 2024^3,4^. This reflects an ongoing increase in cholera outbreaks globally, driven by factors such as poor sanitation, limited access to clean drinking water, conflict zones, and climate-related disruptions. These trends emphasize the urgent need for novel therapeutic strategies to address cholera infections and mitigate its public health impact.

*Vibrio cholerae* remains a global public health challenge, particularly in regions with poor sanitation and limited access to clean water^1^. While conventional treatments focus on antibiotics and oral rehydration, increasing antibiotic resistance emphasizes the need for novel drug targets^5,6^. Recent studies have highlighted respiratory chain complexes in bacteria as promising therapeutic targets due to their essential roles in energy metabolism and ion homeostasis^7,8^. In this context, sodium-pumping NADH-ubiquinone oxidoreductase (Na⁺-NQR) represents a critical membrane-bound enzyme unique to many pathogenic bacteria, including *V. cholerae*, but absent in humans, making it an attractive and selective target for antimicrobial drug discovery^8–10^. This structural and functional divergence provides a high degree of selectivity, minimizing potential off-target effects and cytotoxicity in humans, and makes Na⁺-NQR an attractive antibacterial drug target. The present study aims to explore this target using computational approaches for identifying potential inhibitors.

NADH ubiquinone oxidoreductase (UQ) or Na- NQR is a major ion transport enzyme and the first metabolic enzyme of the respiratory chain of several pathogenic organisms like *Vibrio alginolyticus*, *Vibrio cholerae*, *Haemophilus influenzae* and sodium ions. It is also the first enzyme of the respiratory chain. Moreover, the Na+-NQR from *Vibrio cholerae*, a human pathogen, was found to be a systematic inhibitor of the ubiquinone oxidoreductase, according to a study. This enzyme works as major NA + pump, has wide distribution among pathogenic species, plays a role in energy metabolism, and is absent in the eukaryotic cell^11^. Therefore, it is a crucial target for antibiotic drugs^11–13^. It is determined by the nqr operon that the Na+-NQR is composed of six subunits, which are denoted by the letters NqrA through F. The Na+-NQR has a total molecular weight of approximately 200 kDa. Additionally, it is composed of five redox cofactors, which come in the form of riboflavin, FAD, an a2Fe-2 S cluster, and two FMNs that are covalently bound^14^. Using a sodium ion pump, this enzyme pair is responsible for the transfer of electrons from NADH to UQ, which results in the generation of an electrochemical sodium ion gradient across the inner membrane of the bacterial cell. In the prior study, the process of electron transfer is reported to take place through a pathway that includes at least five redox cofactors, specifically NADH to FAD, a 2Fe-2 S centre, two FMNs that are covalently linked, and a riboflavin, which eventually leads to the arrival at UQ. This is the consensus that has been recognised^7,15^. However, the specific mechanism responsible for the mechanism whereby Na + ions are transported through electron transfer has not even been determined. In addition, Na+-NQR enzyme is also thought to be a more selective antibiotic target than those previously investigated. This is due to the fact that it is only found in prokaryotic organisms and that it does not share any structural similarities with mitochondrial respiratory complex I^16^. Furthermore, under anaerobic conditions, the gene that encodes EQR is put into a state of strong repression. Consideration of the fact that some zones of the small intestine are anaerobic means that the lack of Na+-NQR could not negatively influence the pathogenicity of the bacterium *V. cholerae* O1. However, another prior study show that Na+-NQR is a vital factor in the colonization of the small intestine of mice by *V. cholerae* O1 and the acid tolerance response or ATR^17^. Based on these findings, it appears that Na+-NQR plays a significant role in the virulence of *V. cholerae* O1 and has the potential to serve as a molecular target for the development of any new treatment for cholera. Na⁺-NQR plays a pivotal role in maintaining sodium ion gradients and driving key physiological processes in *V. cholerae*, including motility, nutrient uptake, and pH regulation^8,10^. Its absence in mammalian systems where Complex I (proton-pumping NADH: ubiquinone oxidoreductase) operates instead, it reduces the risk of off-target effects, enhancing its druggability profile^9^. Inhibiting Na⁺-NQR disrupts bacterial bioenergetics, ultimately impairing survival and pathogenicity, as demonstrated in both genetic knockout and small molecule inhibition studies^8,18^. This makes Na⁺-NQR a highly promising target for antimicrobial drug development against *V. cholerae*. Over this, the Na+-NQR (Na+-translocating NADH: ubiquinone oxidoreductase) enzyme was selected as the target in the current investigation^11^.

Previous studies have identified several small-molecule inhibitors targeting Na⁺-NQR, notably aurachin D-42 and korormicin A^19–21^. Aurachin D-42 exhibits potent inhibition against *Vibrio cholerae* Na⁺-NQR (IC₅₀ ≈ 2 nM), binding at the N-terminal region of the NqrB subunit as confirmed by structural and photoaffinity labeling studies^22,23^. Korormicin A, a quinone analogue produced by *Pseudoalteromonas* species, selectively inhibits Na⁺-NQR by blocking ubiquinone reduction at the NqrB subunit, thereby disrupting the electron transport chain^20^. This inhibition not only interferes with bacterial respiration but also promotes reactive oxygen species (ROS) generation, enhancing bactericidal activity^21^. Due to the rising emergence of antibiotic-resistant strains, the discovery of novel Na⁺-NQR inhibitors remains critical.

Traditional Indian and Chinese medicine has used berberine, an alkaloid found in *Berberis aristata* (barberry), as an antidiarrheal. Berberine has been demonstrated to decrease the secretion of *Vibrio cholerae’s* heat-labile enterotoxins. Berberine reduced these secretory responses by 70% in the rabbit ligated intestinal loop model^24^. This inhibiting activity justifies the clinical use of berberine-containing plants to treat acute diarrheal illnesses like cholera. Bacteriostatic and bactericidal activity against *Vibrio cholerae* has also been demonstrated with berberine hydrochloride, suggesting that it may be effective against cholera infections^25^. Antimicrobial, antioxidant, and anti-inflammatory properties of the alkaloid may make it a possible treatment for many disorders. The *Berberis vulgaris* and *Hydrastis canadensis* was selected for this cholera-focused study. This was based on their engrained antimicrobial properties. Berberine, an active compound found in barberry (*Berberis vulgaris*), has showed array of diverse biological activities, which includes antimicrobial, anti-inflammatory, antidiabetic, and antioxidant^26^. In a similar manner, the antibacterial properties of goldenseal, scientifically known as *Hydrastis canadensis*, can be primarily described to its substantial berberine content^27^. It is widely known that these plants possess antibacterial characteristics, particularly in regard to different types of pathogens that can be found in the gastrointestinal tract. Moreover, berberine, that is present in both plants, has been used in traditional Chinese and Ayurvedic medicine, demonstrating its broad-spectrum effectiveness in treating various ailments^28^. The alkaloid’s has a capacity to inhibit various pathogens, including *H. pylori* bacteria, indicates a possible application in fighting against the *Vibrio cholerae*, the etiological agent of cholera^29^.

Therefore, in this current study computational based drug discovery methodologies were used to analyse the therapeutic potential of validated natural products in order to address the essential need for effective cholera treatments. Here, the aim of the study is to discover and enhance natural extracted compounds that have the capacity to suppress the cholera toxin. While previous studies have primarily focused on synthetic inhibitors such as aurachin and korormicin, the present study adopts an integrated in silico approach combining natural product-based virtual screening, machine learning-based activity prediction, ADMET profiling, DFT calculations, and molecular dynamics simulations. This multi-layered strategy enables the identification of novel phytochemical scaffolds with potential Na⁺-NQR inhibitory activity, offering a broader chemical diversity and minimizing the risk of cytotoxicity through selective targeting.

## Methodology

### Data retrieval

The protein structure of *Vibrio cholerae* Na⁺-pumping NADH-ubiquinone oxidoreductase retrieved from Protein Data Bank database (PDB ID: 7XK7) was selected for this study based on its experimental quality and biological relevance^14,30^. This structure was solved using Cryo-Electron Microscopy (Cryo-EM) at a resolution of 2.90 Å, providing a high-quality structural model with complete representation of all six protein subunits (NqrA–F). Importantly, the structure includes the co-crystallized inhibitor korormicin bound at the interface of chains A and B, which allowed accurate identification of the ligand-binding site. The presence of this reference ligand facilitated precise definition of the docking grid for virtual screening. Residues located within an 8 Å radius around korormicin were selected to construct the grid box, ensuring appropriate targeting of the biologically relevant active site involved in sodium translocation and ubiquinone reduction. This interface is critical for enzymatic function and has been previously reported as a key site for inhibitory interactions.

Moreover, in the present study Korormicin was co-crystallized with this protein structure was used as a control. Aurachin D was also found to be known inhibitor of the protein^16,22^; thus, it was also retrieved by the PDB ID: 7XK6 from the PDB. The binding site of both the known inhibitors were identified using these two structures and used for molecular docking.

### Compound library

The compound library for virtual screening was constructed from phytochemicals derived from *Berberis vulgaris* and *Hydrastis canadensis*, selected based on their ethnopharmacological relevance and reported antimicrobial properties. Both plants have been traditionally used for centuries in herbal medicine to treat gastrointestinal infections, including diarrhea, dysentery, and cholera-like symptoms^31,32^. Berberis vulgaris is a rich source of protoberberine alkaloids such as berberine, palmatine, and jatrorrhizine, which exhibit broad-spectrum antimicrobial, anti-biofilm, and efflux pump inhibitory activity against Gram-positive and Gram-negative bacteria^33,34^. Berberine has also demonstrated the ability to interfere with bacterial energy metabolism, suggesting potential interaction with respiratory chain components^35^. Likewise, *Hydrastis canadensis* (goldenseal) contains isoquinoline alkaloids such as hydrastine, canadine, and berberine, which exhibit antibacterial, anti-inflammatory, and quorum sensing inhibitory properties^27,36^. The inclusion of these phytochemicals allowed us to assemble a focused natural product-based library enriched in bioactive alkaloids with potential antibacterial activity targeting Na⁺-NQR in *Vibrio cholerae*.

Phytochemicals from Berberis vulgaris and Hydrastis canadensis were retrieved from the Natural Product Activity and Species Source Database (NPASS)^37,38^ (https://bidd.group/NPASS/index.php). Experimental characteristics and bioactivity data were extracted and preprocessed for compatibility with cheminformatics analysis tools. Clustering of compounds retrieved from the NPASS database was performed to reduce structural redundancy and ensure a representative selection of compounds for virtual screening. Clustering was based on physicochemical features derived from Lipinski’s Rule of Five descriptors (molecular weight, hydrogen bond donors/acceptors, and logP), with molecular fingerprints generated through RDKit. Structurally similar compounds were grouped, and representative members from each cluster were selected. Given the shared biosynthetic origins of the compounds from *Berberis vulgaris* and *Hydrastis canadensis*, some structural similarity is expected. This is particularly true for compounds derived from the same plant family, as they often share core scaffolds and functional groups, which can lead to clustering of similar compounds. This approach ensured that the final compound set maintained a balance between structural variety and computational efficiency during virtual screening. This approach ensured that the final compound set maintained a balance between structural variety and computational efficiency during virtual screening. RDKit^39^ was employed for molecular fingerprint generation, and unsupervised clustering was performed using the scikit-learn (sklearn) library^40^. Initially, several clustering algorithms were tested. The final clustering was performed using hierarchical agglomerative clustering based on molecular fingerprints, while logistic regression was subsequently applied as a classification tool to evaluate and refine cluster assignments based on compound activity profiles. The cluster exhibiting the highest internal consistency and bioactivity relevance was selected for further investigation. The compounds from this cluster were then subjected to virtual screening through molecular docking.

### Virtual screening

The protein structure of Na+-pumping NADH-ubiquinone oxidoreductase was prepared using AutoDock Tools (AutoDock 4.2) to ensure accurate input for docking simulations^41^. Only chains A and B of Na⁺-NQR were retained for molecular docking, as the native ligand korormicin binds at the interface of these two subunits, forming the primary binding pocket. The remaining subunits (C–F), which are not directly involved in ligand binding, were excluded to simplify the docking system while maintaining biological relevance. All non-standard residues, heteroatoms, and crystallographic water molecules were removed. Polar hydrogens were added, and Kollman charges were assigned to accurately model electrostatic interactions during docking. Ligand structures were geometry-optimized, energy minimized, and converted into pdbqt format for compatibility with AutoDock Vina.

The docking grid was defined around the binding pocket of Korormicin, a known co-crystallized inhibitor of Na+-pumping NADH-ubiquinone oxidoreductase, to ensure that the active site was accurately targeted. The grid box was centered at coordinates (121.952, 162.319, 152.236) with dimensions of 30 Å × 30 Å × 30 Å, covering all residues within an 8 Å radius of the reference ligand. This setup allowed for complete sampling of the binding pocket and ensured inclusion of key interacting residues.

Molecular docking was performed using AutoDock Vina^42^. The docking exhaustiveness was set to 500 to enhance sampling accuracy, num_modes was set to 20 to explore multiple potential binding conformations, and the energy range was set to 4 kcal/mol to retain poses within this energetic window relative to the best score. An exhaustiveness value of 500 was chosen to ensure comprehensive sampling of the binding site due to the flexible nature of the ligand-binding pocket. A grid spacing of 1 Å was used to maintain fine resolution of the search space. The AutoDock Vina scoring function was employed to predict binding affinities based on the calculated interaction energies. The virtual screening allowed for systematic evaluation of phytochemical compounds against the target binding site, and several top-ranked candidates were selected for further binding energy estimation and dynamic simulation analysis.

### Calculation of LE and Ki

To further evaluate the binding characteristics of the docked complexes, ligand efficiency (LE) and estimated inhibition constants (*K*_*i*_) were calculated based on the docking binding energies. Ligand efficiency was calculated by dividing the docking binding free energy (*ΔG*, in kcal/mol) by the number of heavy atoms (non-hydrogen atoms) in each ligand. The number of heavy atoms was determined using SwissADME (http://www.swissadme.ch/)^43^. The estimated *K*_*i*_ values were calculated using the standard thermodynamic relationship between binding free energy and inhibition constant^44^:

$$\:\varDelta\:G=\:RT\text{l}\text{n}{K}_{i}$$

where R is the gas constant (1.987 cal/mol·K), T is temperature (298 K), and *ΔG* is the docking binding energy converted to cal/mol. The resulting *K*_*i*_ values were expressed in nanomolar (nM) units. All calculations were performed using custom Python scripts which were deposited in a publicly accessible repository (Zenodo, 10.5281/zenodo.16835099) for transparency and reproducibility.

### Electrostatic surface potential calculation

Electrostatic surface potential calculations were performed using the Adaptive Poisson–Boltzmann Solver (APBS)^45^ integrated into PyMOL^46^. The protein-ligand complexes, including the control complex and the top docked complexes, were prepared for electrostatic calculations by first assigning atomic charges and radii using PDB2PQR (v3.6.1) (https://server.poissonboltzmann.org/pdb2pqr)^47^ with the CHARMM force field. The generated PQR files were loaded into PyMOL, and electrostatic potential maps were computed using APBS with optimized grid parameters to ensure successful convergence. The electrostatic potential was mapped onto the molecular surfaces and visualized using a color gradient ranging from − 5.0 kT/e (red) to + 5.0 kT/e (blue), representing negative and positive electrostatic potentials, respectively.

### Interaction profile and ADMET

An ML-based predictive model, PSICHIC (PhySIcoCHemICal graph neural network) (https://psichic.dcmb.med.umich.edu/)^48^, was employed to analyse the binding affinity and interaction properties of the selected compounds. PSICHIC server employs a graph neural network (GNN)-based architecture that model’s protein–ligand interactions by interweaving intra- and intermolecular features through three physicochemical graph convolutional layers. At each layer, PSICHIC passes messages between atoms within the ligand and between residues within the protein using two independent GNNs, allowing it to capture both local and global interaction patterns. This model directly decodes ligand-receptor interactions from sequence data. This was supplemented by ADMET profiling to assess pharmacokinetic properties. The physicochemical and ADMET (Absorption, Distribution, Metabolism, Excretion, and Toxicity) properties computed using SwissADME (http://www.swissadme.ch/), the ProTox-3.0 server (https://tox.charite.de/protox3/index.php?site=home)^49^ and pkCSM (http://biosig.unimelb.edu.au/pkcsm/)^50^.

### DFT

Density Functional Theory (DFT) was used to analyse the top three compounds found by molecular docking. One common approach to ab initio calculations at the atomic, molecule, crystal, surface, and interaction levels is density functional theory^51^. When it comes to quantum chemistry, DFT is by far the most used theoretical method^52^. The selection of the B3LYP functional combined with the 6-31G* basis set for Density Functional Theory (DFT) calculations is a well-established practice in computational chemistry, particularly for studies involving organic molecules. This combination has been extensively validated and is known for providing a good balance between computational efficiency and accuracy. A previous study evaluated the performance of B3LYP with various basis sets, including 6-31G(d), across 622 organic compounds. They reported mean absolute errors (MAEs) in heats of formation ranging from 2.4 to 3.1 kcal/mol, demonstrating the reliability of this method for organic systems^53^. While more advanced functionals and basis sets, such as M06-2X or 6–311 + + G**, can offer improved accuracy for specific systems, they also come with increased computational costs^54^. Therefore, the B3LYP/6-31G* level of theory remains a practical and validated choice for initial geometry optimizations and electronic property calculations in studies focusing on organic molecules.

A Python program was used to execute the density-functional theory (DFT) calculations for all of the compounds, and the PySCF framework, which is based on Python, was used to conduct electronic structure studies^55^. We used the B3LYP functional, a mix of the exact Hartree-Fock exchange and the DFT exchange-correlation term^53^ and the 6-31G* basis set^56^, to analyse the whole geometry. All DFT calculations were performed in the gas phase (vacuum) to optimize molecular geometries and compute frontier molecular orbital energies without solvent effects. The dipole moments, total energies, and energy levels of the highest occupied molecular orbital (HOMO) and lowest unoccupied molecular orbital (LUMO) were also calculated.

### Molecular dynamics (MD) analysis

Molecular dynamics (MD) simulations were performed to evaluate the flexibility and stability of the protein-ligand complexes. The compound with the highest binding score and the control were selected for this analysis. Due to the structural complexity of the Na-NQR enzyme, which comprises multiple membrane-spanning subunits (NqrA–F) and multiple redox cofactors (FAD, FMNs, Fe-S cluster, riboflavin), full membrane-embedded molecular dynamics (MD) simulations were not performed in the present study. Incorporating the membrane bilayer and parameterizing all cofactors would require extensive system preparation and specialized force field development, which remain technically challenging. Instead, simulations focused on the isolated ligand-binding domain in aqueous solution to directly assess ligand binding interactions and energetics. This approach allowed the evaluation of ligand binding behavior while minimizing system size and computational complexity, providing meaningful insights into ligand-protein interactions at the binding interface.

Molecular dynamics (MD) simulations were performed using GROMACS version 2021.2. The CHARMM36-jul2021 force field package was downloaded from the official CHARMM-GUI repository and integrated into GROMACS. Within this force field, the CHARMM27 parameter set was selected for protein parameterization^57–62^. Although the cgenff server is widely recognized for providing reliable parameters for drug-like molecules, SwissParam was selected in this study due to its compatibility with the CHARMM force field and its broader parameter coverage for structurally diverse natural product scaffolds^63^. Direct comparison with cgenff was not performed, as some phytochemical scaffolds lacked coverage in cgenff. Furthermore, the Particle mesh Ewald (PME) approach^64^ was implemented in the electrostatic force computation. To investigate the adaptability and stability of protein-ligand complexes, the compounds that exhibited the highest levels of activity were selected along with the control group. The TIP3P water model was employed for system solvation as it is fully compatible with the CHARMM force field, which was originally parameterized using this model^65^. Although higher-order models like TIP4P or TIP5P better reproduce certain thermodynamic properties of bulk water, TIP3P remains widely adopted in CHARMM-based protein-ligand and membrane protein simulations due to its compatibility and reliable performance in reproducing binding energetics^66,67^. Moreover, previous studies have shown that employing TIP3P in combination with accurate protein or membrane composition may yield more realistic simulation results compared to using TIP4P with incomplete system parameterization^68^. A dodecahedron was used as a structure for the simulation of all complexes, and the buffer distance among them was set at 1 Å. Over the course of 5000 iterations, in order to bring about a decrease in the energy of the protein-ligand coupled complex, the steepest descent (SD) strategy was applied. The system achieves stability by increasing the temperature to 310 K and removing all the hydrogen bonds by employing the LINCS algorithm^69^. All solvated protein-ligand complexes were subjected to energy minimization using the steepest descent algorithm in GROMACS for 5000 steps to eliminate steric clashes and optimize system geometry prior to molecular dynamics simulations. Later, the system endures a homogenous ensemble with constant temperature (NVT) conditions for a duration of 1 ns at 310 K and 1 atmosphere, respectively, to attain equilibrium. Subsequently, this production run, which lasted for one hundred nanoseconds, was carried out using the equilibrated system. In this instance, the Parrinello-Rahman pressure method^70^ was employed to maintain a constant pressure, whereas the velocity-rescaling method^71^ was applied with the goal of coordinating the temperature. An investigation was conducted using the GROMACS built-in tool to examine the RMSD, RMSF and hydrogen bonds.

### PCA and FEL

Principal component analysis (PCA) was accomplished on protein-ligand complexes using GROMACS^57,58^ GROMACS is a powerful molecular dynamics simulation tool, with the default settings. The trajectory of these protein-ligand complexes was initially processed to remove periodic boundary conditions before performing PCA for more accurate analysis. The GROMACS ‘gmx covar’ function calculated the protein-ligand complex’s covariance matrix, which shows atomic variations. This matrix is crucial for understanding complex dynamics. Later, it was the ‘gmx anaeig’ tool that was used in calculating eigenvalues and eigenvectors of the covariance matrix. This was an essential finding for the system’s main molecular dynamics components that explain most of the variance. For projecting and visualising trajectory data to project onto those of such principal components, a GROMACS utility ‘gmx anaproj’ was employed, and a subsequent calculation of PC coordinates for every frame of the trajectory.

The free energy landscape was also used to study protein structural changes and energy states^72^. Additionally, FEL analysis enables the researcher to comprehend the biomolecules’ recognition, processing, and folding. Moreover, to calculate the free energy landscape, by using the equation ΔG(X) = -k_BT ln P(X), in this equation, the variable ΔG represents the change in Gibbs free energy, k B symbolises the Boltzmann constant, T represents the absolute temperature, representing the reaction coordinate, and P(X) represents the probability distribution function. This method was used to analyse the protein-ligand complex’s stable and transient states, characterized by FEL minima and barriers. This strategy provided a complete vision of the molecular dynamics and energetics of our protein-ligand complexes.

### MM/GBSA

The gmx_MM/PBSA plugin of GROMACS was used to estimate the binding free energy of the protein-ligand complex in dimer form at the end of 20 ns of the simulation^73,74^. The method applied in the study includes multiple thermodynamic factors to compute the binding free energy of the complex. The binding free energy (∆G_*binding*_) was measured using a set of mathematical equations described below:

1 $${\rm \:\Delta}\:G={G}_{complex\:\:}-[\:{G}_{receptor}+\:{G}_{ligand}]$$

2 $$\:{{\Delta\:}\text{G}}_{binding}\:=\:{\Delta\:}\text{H}\:-\:\text{T}{\Delta\:}\text{S}$$

3 $$\:{\Delta\:}\text{H}\:=\:{\Delta\:}{\text{G}}_{\text{G}\text{A}\text{S}}\:+\:{\Delta\:}{\text{G}}_{\text{S}\text{O}\text{L}\text{V}}$$

4 $$\:{\Delta\:}{\text{G}}_{\text{G}\text{A}\text{S}}=\:{\Delta\:}{\text{E}}_{\text{E}\text{L}}\:+\:{\Delta\:}{\text{E}}_{\text{V}\text{D}\text{W}\text{A}\text{A}\text{L}\text{S}}$$

5 $$\:{\Delta\:}{\text{G}}_{\text{S}\text{O}\text{L}\text{V}}\:=\:{\Delta\:}{\text{E}}_{\text{G}\text{B}}\:+\:{\Delta\:}{\text{E}}_{\text{S}\text{U}\text{R}\text{F}}$$

6 $$\:{\Delta\:}{\text{E}}_{\text{S}\text{U}\text{R}\text{F}}\:=\:{\upgamma\:}.\text{S}\text{A}\text{S}\text{A}$$

Here, in this approach, SASA was used to represent the SASA of the molecule, and γ to represent the surface tension of the solvent. The symbols ∆E_*VDWAALS*_ and ∆E_*EL*_ represent the variations in van der Waals and electrostatic energies, respectively. The variables ∆E_*GB*_ and ∆E_*SURF*_ signify the variations in solvation energies by relating to polar and nonpolar substances, respectively. The application of this inclusive methodology enables a precise assessment of the binding free energy of the complex, providing valuable observations concerning the molecular interactions and stability of the complex. A significant feature of g_mmpbsa and its ability to evaluate the energy influence per residue on the binding energy, allowing for the identification of critical residues in protein-ligand interactions^75^. It was conducted to determine the role of the residues in the binding of the protein.

### Network Pharmacology

To identify potential protein targets of the selected compounds, the DIGEP-Pred webserver (http://www.way2drug.com/GD/) was utilized, using a pharmacological activity threshold of 0.5, the CTD_mRNA dataset, and selecting only down-regulated targets^76^. The predicted protein targets were subsequently analyzed through the STRING database (https://string-db.org/) for protein-protein interaction validation and optimization^77^. Functional enrichment analysis was performed using the Enrichr database (https://maayanlab.cloud/Enrichr/) to categorize associated biological processes, molecular functions, cellular components, and KEGG pathways. For interaction network construction and visualization, the NetworkX library in Python was employed^78^. NetworkX was specifically chosen for its seamless integration within the existing Python-based computational pipeline, allowing automated network generation, analysis, and figure production without requiring external software, while Matplotlib was used for graphical representation.

## Results

### Protein structure

**Fig. 1:**
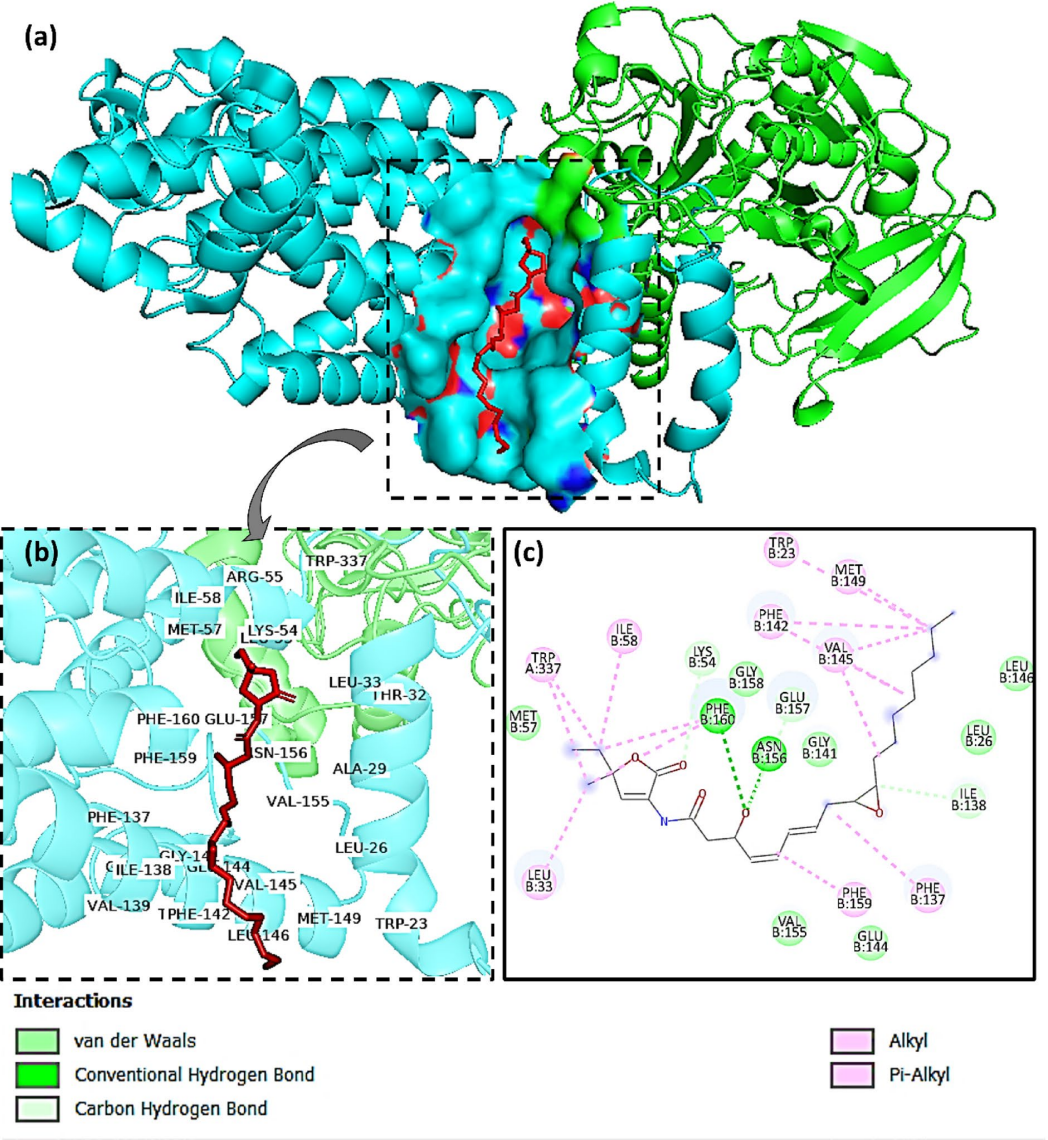
(**a**) Korormicin bound to the Chain A and B of Na+-pumping NADH-ubiquinone oxidoreductase, (**b**) residues around Korormicin (8 Å) with the protein, (**c**) interacting residues with Korormicin.

In this study, NADH-ubiquinone (UQ) oxidoreductase, which is responsible for the Na+-pumping, was examined specifically. The protein is a hexameric complex consisting of six unique chains that are co-crystallized with korormicin. Korormicin was observed to bind to chain B of the protein structure and also interact with a portion of chain A, as depicted in Fig. 1(a). Biovia Discovery Studio^79^ revealed that korormicin formed several interactions with residues in chain B. Additionally, it formed a bond with a single residue (Trp337) in chain A, as depicted in Fig. 1(b). Therefore, both chain A and chain B were isolated for molecular docking along with korormicin for subsequent analysis. Furthermore, a prior study demonstrated that the inhibitors disrupt the UQ process by obstructing the interface between chain A and B of the protein^16^. Additionally, the residues that interact with korormicin within a distance of approximately 8 Å were used to construct the grid box for the docking process, as depicted in Fig. 1(b). As shown in Fig. 1(c), korormicin forms carbon-hydrogen bonds with residues Ile138 and Gly157, as well as conventional hydrogen bonds with Asn156 and Phe160 of chain B, highlighting key interactions within the binding pocket. The essential residues are likely engaging with the established inhibitor korormicin. Aurachin D was identified as a protein inhibitor. The binding location of the known inhibitors was determined utilising these two structures and subsequently utilised for molecular docking.

### Compound library

Phytochemicals from each of the plant species, *Berberis vulgaris* and *Hydrastis canadensis*, were repossessed from the Natural Product Activity and Species Source Database (NPASS). Here, 20 phytochemicals were found from *Berberis vulgaris* and 23 phytochemicals from *Hydrastis canadensis*. A total of 43 phytochemicals were used further for investigation, as shown in the supplementary Table S1.

The 43 phytochemicals from *Berberis vulgaris* and *Hydrastis canadensis* were retrieved from the NPASS database and subjected to clustering using a logistic regression model implemented via the sklearn module in Python. Clustering was performed based on physicochemical features derived from Lipinski’s Rule of Five descriptors (molecular weight, hydrogen bond donors/acceptors, and logP). This process produced four distinct clusters, from which the largest, comprising 28 compounds, was selected for downstream virtual screening, owing to its high classification accuracy (0.94).

Although certain structural similarities are present within this cluster, this is expected due to the common biosynthetic origins of the compounds and the characteristics of the physicochemical attributes employed for grouping. The clustering process prioritized drug-likeness based on these features rather than purely structural diversity, which may have resulted in the inclusion of compounds with similar scaffolds. Thus, the cluster with the highest population, with 28 compounds, was used for virtual screening along with the two controls.

### Virtual screening

Post clustering, 28 phytochemicals were virtually screened against the target protein. The functionality of chains A and B of the protein has been used for the molecular docking. Two controls, Korormicin and Aurachin D, were also docked in the same binding pocket with the same protocol. The compounds that exhibited favourable hydrogen bonding and docking scores were selected for additional analysis via MD simulation. As shown in Table 1, the top 15 compounds that showed better performance than the controls were further used for interaction analysis. The top 15 compounds, along with the two controls, had high binding affinity with the binding score in the range − 8 kcal/mol to −9.6 kcal/mol. The 2D structure of the top 15 compounds are shown in the supplementary Figure S1.

The Tanimoto similarity heatmap, as shown in supplementary Figure S2, quantitatively illustrates the diversity of the selected compounds. While certain compounds exhibit higher similarity (values closer to 1), there is still a wide range of Tanimoto similarity values, from as low as 0.06 to 1.00. This variation demonstrates that although some compounds share core structural features, significant differences exist across the selection. Compounds with low similarity scores (< 0.2) remain in the final compound set, ensuring diversity in their chemical scaffolds. This clustering method, which uses Lipinski’s Rule of Five descriptors along with molecular fingerprints, maintains a balance between structural variety and computational efficiency for virtual screening. In addition, while clustering prioritized drug-likeness based on physicochemical properties, the heatmap provides further evidence of the structural diversity, especially when considering the similarity between the controls and selected compounds. This approach ensures that despite some compounds being structurally similar, the overall diversity and representativeness of the library are maintained.

**Fig. 2:**
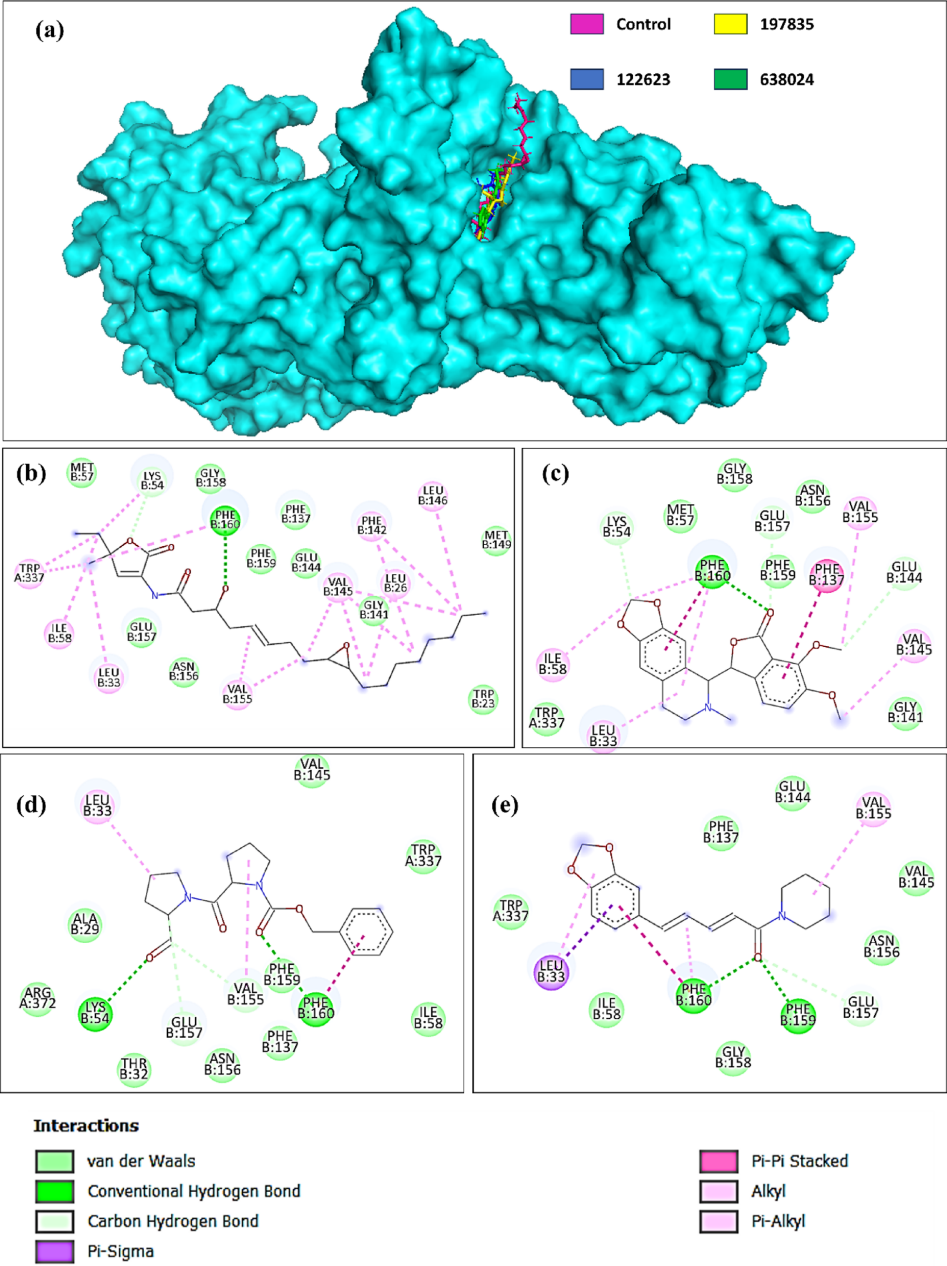
(**a**) 3D representation of the interaction between protein and ligand, and 2D representation of interaction analysis for the compounds (**b**) Control (Korormicin), (**c**) 197835, (d) 122623, (e) 638024.

It was found that among the two controls, with the residues Lys54 and Phe160 of the chain B, korormicin was observed to be have two hydrogen bonds, as can be seen in Fig. 2(b). However, Aurachin D was not found to have hydrogen bonds; thus, it was not used further for investigation. Out of the top 15 compounds, exhibited hydrogen bonds with the protein residues; however, compounds **197835**, **371942**, **122623**, and **638024** showed the maximum number of hydrogen bonds, as illustrated in Fig. 2. Here, **197835** and **371942** showed identical interaction and the same binding score of −9.6 kcal/mol; however, **197835** showed a lower average binding score than **371942**.

The structures of the top 15 docked compounds were analyzed and are presented in the revised Table 1. Among them, compounds **197835** and **371942** share identical atom connectivity but differ in stereochemistry, as confirmed by InChI analysis shown in supplementary Table S2. Specifically, **197835** has both stereocenters defined with configurations at C18 (−) and C19 (+), while **371942** has one stereocenter (C18) unspecified and the other (C19) with the opposite configuration (−). Despite these stereochemical differences, both compounds exhibit identical docking scores (− 9.6 kcal/mol) and interactions due to their shared molecular framework.

The structural comparison in Figure S1 illustrates the stereochemical differences between compounds **197835** and **371942**. Despite these differences, both compounds adopt similar binding orientations within the target’s active site, contributing to their identical docking scores. The images highlight how the stereochemistry at the chiral centers (C18 and C19) of **197835** and **371942** influences their hydrogen bonding patterns, yet the overall molecular framework remains similar.

This explains the reason they show identical binding energy, while their docking poses differ, the overall interactions with the protein target are similar. In the docking results, **197835** forms five hydrogen bonds (Lys54, Gly141, Asn156, Gly158, Phe160), whereas **371942** forms three (Lys54, Glu157, Phe160). The difference in hydrogen bonds arises from the subtle stereochemical variations, particularly the configurations of the chiral centers, which influence the precise orientation of the ligands in the active site. Based on its more favorable interaction profile, **197835** was selected for further studies. In contrast, compound **470416** was excluded due to its lower number of hydrogen bonds (two), indicating weaker interaction with the target site. The 3D representation of the interactions between the protein and these ligands is shown in Fig. 2(a), where all three compounds occupy the same active site as the control. Thus, **197835**, **122623**, and **638024** were selected along with Korormicin as a control for further studying the stability of the complexes.

**Table 1:** Top and average Docking score along with the no. of hydrogen bonds and the residues interacting for the top 15 compounds along with the two controls, Korormicin and Aurachin D.

Ligand	Top binding energy (kcal/mol)	Average binding energy (kcal/mol)	No. of H-bonds	Residues
197835	**−9.6**	**−7.86**	**5**	**Lys54**,** Gly141**,** Asn156**,** Gly158**,** Phe160**
371942	−9.6	−7.83	3	Lys54, Glu157, Phe160
470417	−9.2	−7.88	0	-
470416	−9.1	−7.71	2	Lys54, Gly141
470418	−8.9	−7.78	1	Lys54
21171	−8.9	−7.53	1	Lys54
11066	−8.6	−7.89	1	Lys54
122623	**−8.6**	**−7.37**	**4**	**Lys54**,** Glu157**,** Val155**,** Phe160**
3083788	−8.5	−7.79	1	Lys54
6426334	−8.5	−7.67	0	-
10246509	−8.5	−7.65	0	-
2353	−8.5	−7.61	0	-
44258359	−8.4	−7.69	0	-
443422	−8.4	−7.57	0	-
638024	**−8.3**	−7.34	**3**	**Glu157**,** Phe159**,** Phe160**
ONI (Aurachin D)	−8.2	−7.67	0	-
IQT (Korormicin)	**−8**	**−7.51**	**2**	**Lys54**,** Phe160**

### Ligand efficiency and estimated Inhibition constants

The calculated ligand efficiency (LE) and estimated inhibition constants (Ki) for the control compound and the top docked ligands are summarized in Table 2. Among the analyzed ligands, compound **197835** exhibited the most favorable ligand efficiency (− 0.343 kcal/mol/atom) and the lowest estimated Ki (90.97 nM), suggesting stronger binding affinity relative to the control and other candidates. The control compound **9845752** (Korormicin) displayed the weakest binding with an estimated Ki of 1356.45 nM and the lowest ligand efficiency (− 0.258 kcal/mol/atom). The remaining compounds, 638024 and 122623, demonstrated intermediate binding affinities with estimated Ki values of 817.28 nM and 492.42 nM, respectively. These calculations provide a comparative assessment of the binding efficiencies and potential inhibitory strengths of the identified ligands.

**Table 2:** Calculated ligand efficiency (LE) and estimated Inhibition constant (Ki) for the docked complexes.

Molecule	Heavy atoms	Docking score (kcal/mol)	Ligand efficiency (kcal/mol/atom)	Estimated Ki (nM)
638024	21	−8.3	−0.395	817.28
197835	28	−9.6	−0.343	90.97
122623	24	−8.6	−0.358	492.42
9845752 (control)	31	−8.0	−0.258	1356.45

### Electrostatic surface potential analysis

**Fig. 3:**
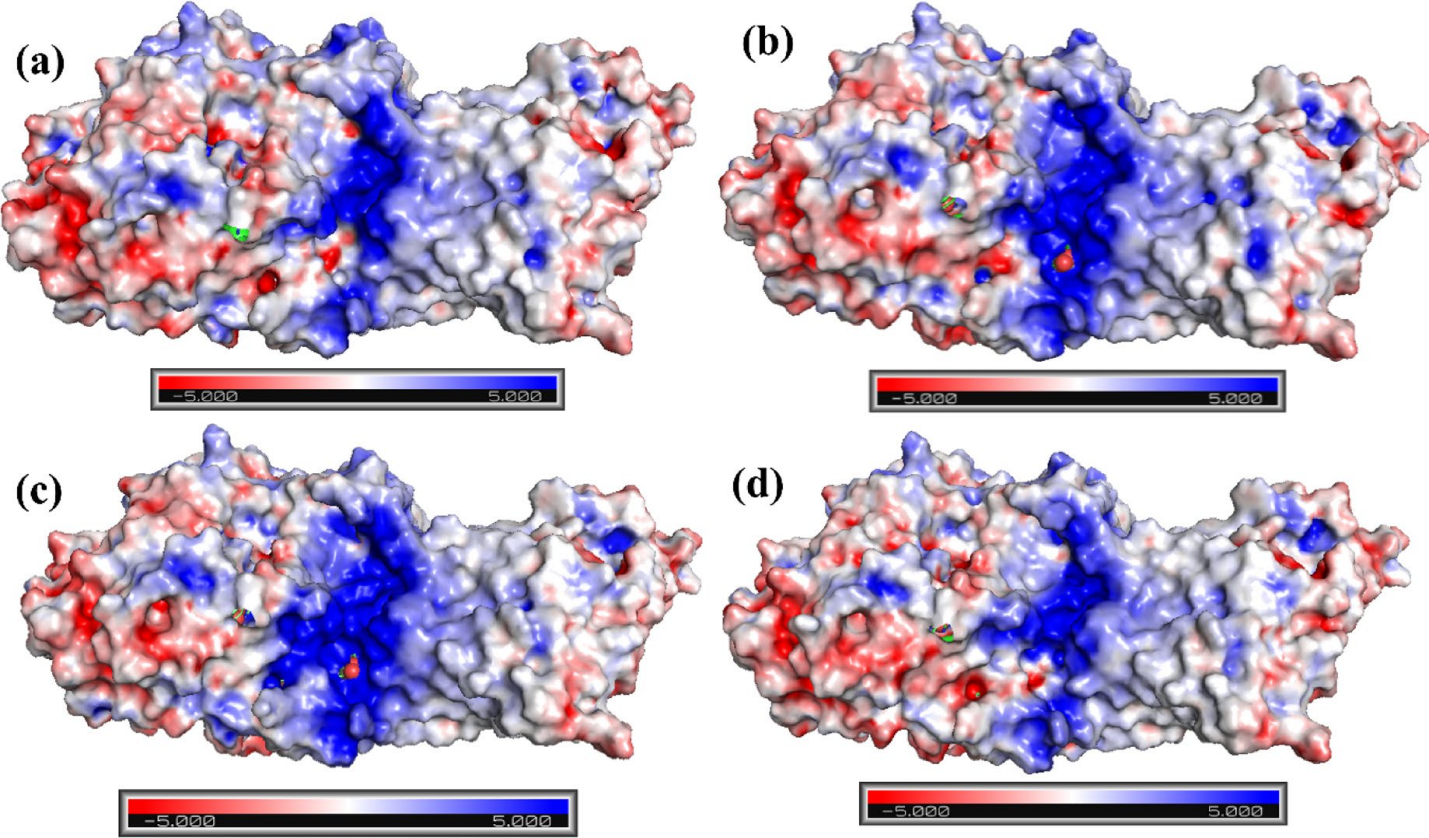
Electrostatic surface potential analysis of the control and ligand-bound complexes generated using APBS in PyMOL. Complex with (**a**) Control (Korormicin), (**b**) 197835, (**c**) 122623, (d) 638024.

Electrostatic surface potential analysis was conducted for the control complex (Korormicin) and the top docked ligand-bound complexes (**197835**, **122623**, and **638024**) using APBS in PyMOL. The molecular surfaces were colored according to the electrostatic potential, ranging from − 5.0 kT/e (red) to + 5.0 kT/e (blue), enabling visualization of charge distribution at the binding interface as shown in Fig. 3. All complexes demonstrated overall positive electrostatic potential at the binding site, reflecting the presence of positively charged residues within the pocket. Compared to the Korormicin-bound complex, complexes **197835** and **122623** exhibited an expansion and intensification of positive potential (increased blue regions), suggesting that these ligands may stabilize additional polar or charged interactions within the binding pocket. Complex 638024, while still maintaining positive potential, displayed a more moderate distribution, closely resembling the electrostatic environment observed in the control complex. These ligand-specific variations in electrostatic surface potential may influence binding interactions, orientation, and stability of the complexes. Overall, these observations suggest that ligand binding induces localized electrostatic rearrangements, which may contribute to binding affinity and specificity.

#### ADMET properties

Three compounds (**638024**, **197835**, and **122623**), along with a control drug, Korormicin, had their physicochemical and ADMET (Absorption, Distribution, Metabolism, Excretion, and Toxicity) properties computed using SwissADME^43^ and the ProTox-3.0 server^49^. Table 3 lists the properties in detail for the four compounds. Here, **638024**, **197835**, and **122623** fall within the range of molecular weights (285.34–330.38.34.38 Da) that are favourable for drug-like compounds (500 Da). The upper drug-like threshold is being approached by korormicin, which has the highest MW at 433.58 Da. The maximum number of rotatable bonds (7) is found in compound **122623**, suggesting that it has greater molecular flexibility. With sixteen rotatable bonds, korormicin is even more malleable. With the exception of korormicin, which has two, all of the compounds have few H-bond donors. Among the many compounds that can serve as H-bond acceptors, compound **197835** has the most, with seven. Korormicin has the highest Molecular Refractivity (MR), which is in line with its higher MW, with a range of 85.47 to 123.21. Drug permeability is indicated by the values of Topological Polar Surface Area (TPSA). The oral bioavailability threshold remains below 140 Å² for all drugs, including Korormicin. The higher TPSA of compounds **122623** and Korormicin indicates that they are more hydrophilic. It was observed that **122623** has an iLOGP (lipophilicity) value of 2.75, while Korormicin has an iLOGP (lipophilicity) value of 4.43. All the compounds showed good solubility. High gastrointestinal absorption indicates good oral availability for all drugs, including the control. A score of 0.55 indicates that all compounds have moderate bioavailability. It showed that these compounds do not include any substructures that are known to cause false positives in drug screening, as no PAINS warnings have been detected. Most synthetically accessible compounds are **638024** (2.92) and **122623** (3.33), whereas the least accessible is korormicin (5.67). Compounds **638024**, **197835**, and **122623** are classified as Toxicity Class 4 (acceptable toxicity) according to the Hazard Communication Standard (HCS) of the Globally Harmonised System of Classification and Labelling of Chemicals (GHS). Class 6 toxicity indicates very minimal or no toxicity for korormicin. All of the compounds exhibit drug-like characteristics, including excellent absorption and bioavailability. The bioavailability score predicted by SwissADME was 0.55 for all compounds, as they fulfilled standard drug-likeness criteria. Additional human intestinal absorption (%) values were retrieved from pkCSM to further characterize oral bioavailability. The exceptional solubility, synthetic accessibility, and tolerable toxicity of compounds **638024** and **122623** differentiate them. The compounds outperform the control in several respects, such as solubility and ease of production, as shown by these features.

**Table 3:** Physiochemical properties (ADMET) of the three compounds and the control.

Properties	638024	197835	122623	Korormicin (control)
MW	285.34	383.39	330.38	433.58
Rotatable bonds	4	3	7	16
H-bond acceptors	3	7	4	5
H-bond donors	0	0	0	2
MR	85.47	103.38	95.75	123.21
TPSA	38.77	66.46	66.92	88.16
iLOGP	3.38	3.4	2.75	4.43
Solubility	Soluble	Moderately soluble	Soluble	Moderately soluble
GI absorption	High	High	High	High
Bioavailability Score	0.55	0.55	0.55	0.55
PAINS alerts	0	0	0	0
Synthetic Accessibility	2.92	4.03	3.33	5.67
Toxicity Class	4	4	4	6

### ADMET validation using PkCSM

Pharmacokinetic predictions were evaluated through ADMET profiling using pkCSM,. All compounds demonstrated high intestinal absorption (> 90%), with compound **197835** exhibiting the highest absorption rate among the evaluated ligands. Favorable Caco-2 permeability values were observed for all compounds, with **638024** showing the highest permeability, suggesting efficient intestinal transport. The volume of distribution (VDss) was acceptable across the ligands, with the control exhibiting comparatively lower distribution than the top-ranked compounds. Predictions for blood-brain barrier (BBB) permeability indicated limited CNS penetration for most ligands; however, **638024** and **122623** displayed moderate BBB penetration, whereas the control and **197835** demonstrated lower permeability. Toxicity assessments consistently predicted no AMES mutagenicity for any compound, while hepatotoxicity was observed only for the control, **638024**, and **122623**. The high level of concordance across multiple predictive platforms underscores the favorable pharmacokinetic and toxicity profiles of the selected ligands, particularly highlighting compound **197835** as a promising candidate. A detailed summary of the pkCSM-based ADMET predictions is presented in Table 4.

**Table 4:** pkCSM-predicted ADMET profiles for the control and top docked compounds.

Molecule	Water solubility (log mol/L)	Caco2 Permeability (log Papp)	Intestinal absorption (%)	VDss (log L/kg)	BBB Permeability (log BB)	CNS Permeability (log PS)	AMES Toxicity	Hepatotoxicity
Control	−4.717	0.619	90.781	−0.278	−0.653	−3.048	No	Yes
197835	−3.733	1.145	95.628	0.513	−0.555	−2.814	No	No
638024	−3.841	1.893	94.355	0.268	0.274	−1.625	No	Yes
122623	−3.392	0.935	93.846	0.033	0.218	−2.937	No	Yes

#### Density functional theory (DFT) analysis

In order to compare the electrical characteristics of three compounds (**197835**, **638024**, and **122623**) against the control, density functional theory (DFT) calculations were conducted. The four compounds’ DFT analyses are listed in Table 5. The stability of the compounds is reflected in their total energy. With an energy of −1389.14 Hartree, Korormicin is the most stable of the compound. At −1302.05 Hartree, **197835** is the most stable of the compounds, with **122623** and **638024** following at −1095.42 and − 927.762 Hartree, respectively. Its HOMO energy indicates a molecule’s electron-donating capabilities. Because of its extremely low HOMO value of −0.13367 eV, korormicin is the most unlikely of all compounds to transfer electrons. Because of its high HOMO energy, compound **638024** is more likely to donate electrons than any other (−0.11323 eV). Acquiring electrons is indicated by the LUMO energy, which stands for the Lowest Unoccupied Molecular Orbital. The lowest electron-accepting capability relative to the others is demonstrated by **122623**, which has the highest LUMO energy at 0.074703 eV. Since **638024** (0.038739 eV) and Korormicin (0.057417 eV) have lower LUMO values, they are more effective electron acceptors. A molecule’s polarity can be measured by its dipole moment. The most polar compound in the set is compound **122623**, which has the most significant dipole moment (4.469887 Debye). With a dipole moment of only 1.03579 Debye, korormicin is clearly the least polar of the bunch. Compounds 197835 (3.13322 Debye) and 638024 (3.027657 Debye) exhibit a moderate degree of polarity. Here, **122623** may be more active in polar biological settings due to its extreme polarity, while 197835 achieves equilibrium by its moderate polarity, balanced HOMO-LUMO energies, and satisfactory stability.

The HOMO–LUMO gap, calculated as the energy difference between LUMO and HOMO levels, was also determined to assess the electronic stability and chemical reactivity of the compounds. A lower HOMO–LUMO gap generally indicates higher chemical reactivity and lower kinetic stability, while a larger gap suggests greater stability and lower reactivity. Among the tested compounds, 638024 exhibited the lowest HOMO–LUMO gap (0.152 eV), suggesting relatively higher reactivity compared to the others. In contrast, 122623 and Korormicin displayed slightly higher gaps (0.193 eV and 0.191 eV, respectively), indicating greater electronic stability. The compound 197835 showed an intermediate gap of 0.178 eV, balancing stability and reactivity. These differences may contribute to variations in interaction strength, binding adaptability, and biological activity among the studied compounds. The dipole moment, which correlates with molecular polarity and membrane permeability, was highest for **122623** (4.4699 Debye), indicating higher polarity and potentially reduced membrane permeability. In contrast, Korormicin showed the lowest dipole moment (1.0358 Debye), suggesting better lipophilicity and membrane diffusion, while **638024** and **197835** exhibited moderate dipole moments (~ 3.0 Debye), indicating balanced permeability characteristics.

**Table 5:** Total energy (Hartree), HOMO (eV), LUMO (eV), HOMO–LUMO gap (eV) and dipole moment (Debye) for the three selected compounds and the control as determined by DFT calculations.

Pubchem ID	Energy (Hartree)	HOMO energy (eV)	LUMO energy (eV)	HOMO–LUMO Gap (eV)	Dipole magnitude (Debye)
Korormicin (control)	−1389.14	−0.13367	0.057417	0.191087	1.03579
197835	−1302.05	−0.11751	0.060544	0.178054	3.13322
638024	−927.762	−0.11323	0.038739	0.151969	3.027657
122623	−1095.42	−0.11832	0.074703	0.193023	4.469887

#### ML-based interaction properties

Further, the compounds were used for interaction properties analysis using PSICHIC (PhySIcoCHemICal graph neural network), a machine learning (ML)-based tool specifically designed for protein-ligand interaction prediction^48^. PSICHIC directly decodes interaction signatures from sequence data, utilising physicochemical constraints. It efficiently evaluated the binding affinity, antagonistic/agonistic potential, and nonbinding probabilities of the selected compounds through the implementation of ML-driven predictive modelling. PSICHIC also demonstrated a high level of accuracy in predicting functional effects with a score of 0.96. On large-scale benchmarking against the PDBBind v2016 and v2020 test sets, PSICHIC achieved binding affinity prediction performance with root mean squared error (RMSE) between 1.31 and 1.34 log units and Pearson correlation coefficients ranging from 0.71 to 0.79, demonstrating robust regression performance for binding free energy estimation^48^. Notably, error analyses showed no significant dependence of prediction error on protein structure resolution (*P* = 0.711), further supporting the model’s stability across diverse input qualities. Additionally, experimental validation of model capabilities has been conducted to identify potential pharmaceuticals. Table 6 lists the predicted interaction properties of the three compounds (**638024**, **197835**, and **122623**) and the control compound Korormicin.

The intensity of the interaction with the target protein is represented by the predicted binding affinity (pKD/pKI). Here, it was found that **197835** has the maximum predicted binding affinity (4.81), which suggests that it has a stronger binding potential than Korormicin (4.79) and other compounds. The binding affinity of **122623** (4.60) and **638024** (4.48) is marginally lower. Predicted antagonist denotes the likelihood that the compound will function as an inhibitor of receptor activity. The antagonist probability of Korormicin is 0.08, which is quite similar to that of 197835 (0.09). The antagonist probabilities of **122623** (0.02) and **638024** (0.01) are low, indicating a diminished potential for receptor inhibition. **197835** demonstrated a robust binding potential. The strongest interaction with target proteins is indicated by the maximum binding affinity (4.81). It exhibited moderate antagonist activity (0.09), rendering it a viable candidate for further investigation. The lowest binding affinity (4.48) of **638024** indicates that it has limited efficacy in protein-ligand interactions.

Here, **122623** exhibited a marginally higher binding affinity (4.60) than **638024**, but it was lower than Korormicin and **197835**. It exhibited a decreased antagonist potential (0.02), which in turn reduced the potential inhibitory effects. Korormicin (Control) exhibited a robust interaction with target proteins, demonstrating a binding affinity that was comparable to **197835** (4.79). It exhibited moderate antagonist potential (0.08), which suggests the potential for receptor inhibition. **197835** is the most promising compound as a result of its balanced interaction profile and high binding affinity. An intermediate option is offered by **122623**, which has moderate binding. Korormicin, which functions as the control, exhibits a well-balanced profile and is a dependable reference point. Further, these compounds **638024**, **197835**, and **122623** were used for additional research.

In this study, both docking scores and ML-based PSICHIC predictions showed broadly similar binding affinity trends for the selected compounds. Minor differences between methods may arise due to their distinct algorithms; docking focuses on static binding poses and energetics, while PSICHIC leverages protein sequence embeddings, physicochemical constraints, and learned interaction features to evaluate ligand binding. This study emphasises the effectiveness of ML-based methodologies in accelerating the prediction of drug-target interactions, improving binding affinity calculations, and refining the drug identification process with increased precision and efficiency.

**Table 6:** Predicted binding affinity ((pK_D_/pK_I_)), and predicted antagonist for the three selected compounds and the control.

Pubchem ID	Predicted binding affinity ((pK_D_/pK_I_))	Predicted antagonist
638024	4.48	0.01
197835	4.81	0.09
122623	4.60	0.02
Korormicin (control)	4.79	0.08

#### Molecular dynamics analysis

##### RMSD

**Fig. 4:**
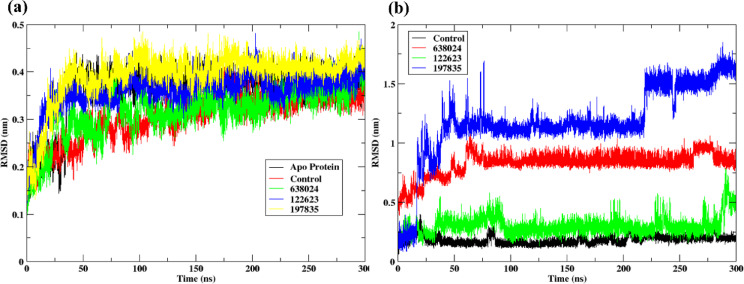
(**a**) RMSD of protein backbone atoms when bound to the ligands and apo form (**b**) RMSD of the ligands, Control (Korormicin), 197835, 122623 and 638024 when bound to the protein for 300 ns.

In addition to the control, the three top-ranked hit compounds were selected for molecular dynamics (MD) simulations. Figure 4(a) presents the backbone RMSD (Cα atoms) of the Na⁺-NQR protein in both apo form and ligand-bound complexes over the 300 ns simulation. The apo protein exhibited higher RMSD fluctuations, stabilizing around 0.35–0.45 nm, indicating greater flexibility in the absence of ligand. Upon ligand binding, the protein RMSD values decreased, demonstrating ligand-induced stabilization of the protein conformation.

Among the ligand-bound systems, the control (Korormicin) and **638024** complexes showed the lowest RMSD fluctuations, stabilizing between 0.2 and 0.3 nm throughout the simulation. The **122623**-bound complex exhibited slightly higher RMSD values (approximately 0.3–0.4 nm) but remained stable after the initial equilibration period. The **197835** complex showed the highest backbone RMSD among all ligand-bound systems, fluctuating around 0.4–0.45 nm, suggesting slightly increased flexibility compared to the control.

The ligand RMSD profiles, shown in Fig. 4(b), revealed distinct stability trends for the ligands during binding. Korormicin and **122623** exhibited the most stable binding modes, maintaining RMSD values below 0.5 nm, indicating strong and consistent interactions with the protein binding pocket. Compound 638024 displayed moderate fluctuations ranging between 0.7 and 1.0 nm, while compound **197835** exhibited the highest ligand RMSD values, reaching up to 1.5 nm. The larger RMSD fluctuations observed for 197835 suggest greater internal flexibility or possible minor rearrangements within the binding cavity during the simulation.

Overall, the results demonstrate that Korormicin and 122623 form stable complexes with Na⁺-NQR, while **638024** and **197835** exhibit higher flexibility. The stable RMSD values of **122623**, comparable to the control, further support its potential as a promising hit compound for targeting Na⁺-NQR. Comparing the two plots, it’s evident that the control provides the most stable interaction with the protein both as a ligand and when the protein is bound to it. Molecules **197835** and **638024** are less stable in both the protein-bound state and as free ligands, as indicated by their higher RMSD values. Overall, the control molecule had the most stable interaction with the protein, which is likely the reason for selecting it as the control. Similar to the control, **122623** could be considered more promising drug candidates due to lower and more consistent RMSD values, indicating a more stable interaction with the target protein. All raw data files, including GROMACS.xvg files, simulation inputs, and processed outputs, were deposited in a publicly accessible repository (Zenodo, 10.5281/zenodo.16835099) for transparency and reproducibility.

### Structural dynamics analysis

**Fig. 5:**
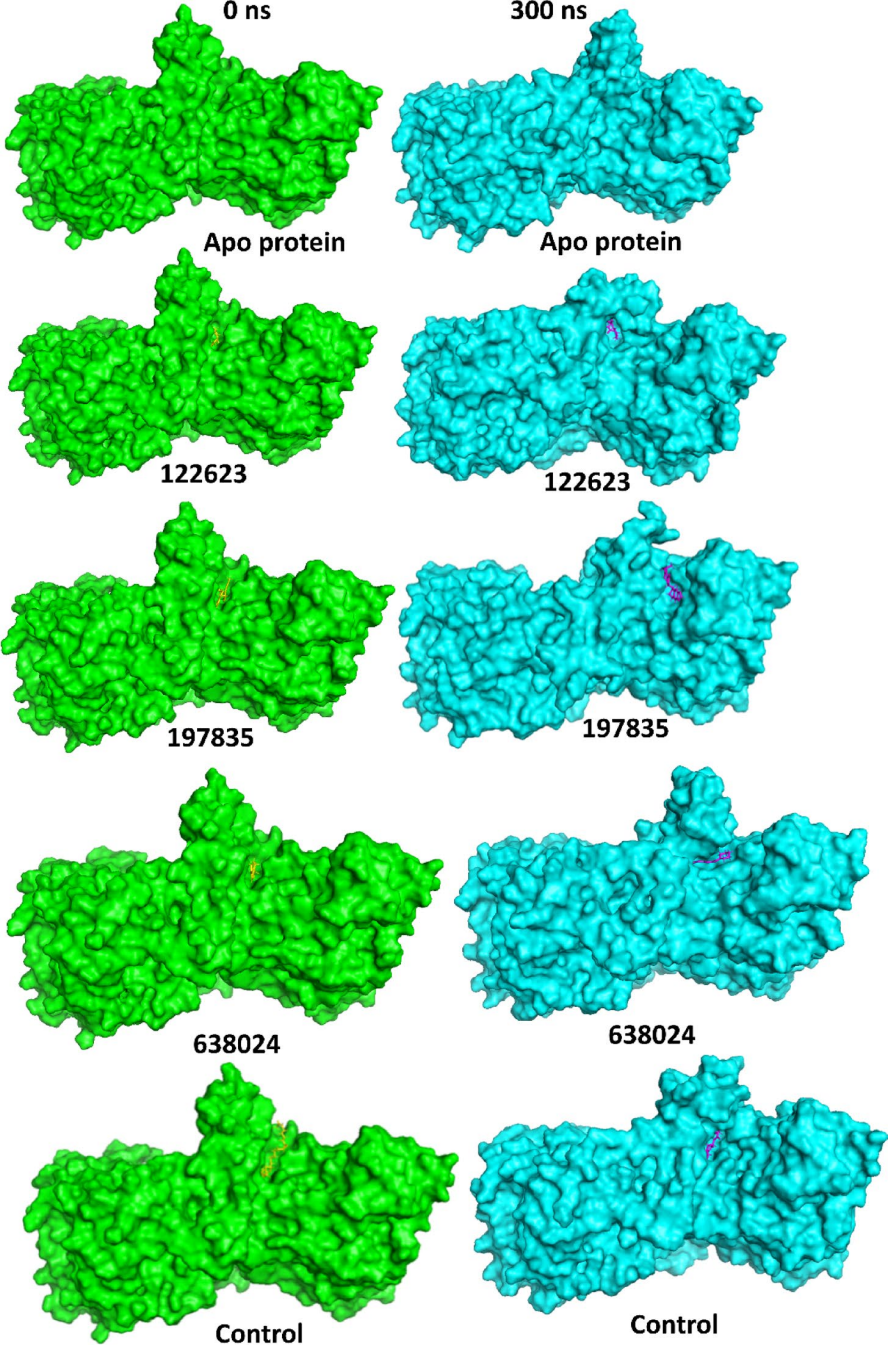
3D conformation of the protein at the 0 ns (initial pose) and 300 ns (final pose) when in apo state and bound to the ligands Control (Korormicin), 197835, 122623 and 638024.

The conformational changes of the Na⁺-NQR protein during the 300 ns molecular dynamics simulations were evaluated for both the apo state and ligand-bound complexes (Fig. 5). The apo protein (green) demonstrated noticeable flexibility and broader structural fluctuations throughout the simulation. In contrast, all ligand-bound complexes (cyan) exhibited relatively stabilized conformations by the end of 300 ns. Among the complexes, 197835 and 122623 displayed more compact and stabilized binding pockets, suggesting stronger protein-ligand interactions that potentially restrict excessive conformational fluctuations. Notably, the complex with 638024 maintained structural integrity but showed slightly greater surface flexibility compared to 197835 and 122623. The ligand binding contributed to stabilizing the active site conformation, as reflected in the reduced global fluctuations compared to the apo structure.

In Fig. 5, the 3D visualizations show clear differences in the protein’s structural dynamics between the initial and final poses. The apo state (green) demonstrates significant structural rearrangements and flexibility, indicative of an unbound, dynamic protein. Meanwhile, the ligand-bound complexes (cyan) exhibit more rigid structures, with 197835 and 122623 inducing tighter and more stabilized binding pockets. 638024, while stabilizing the structure, retains a degree of flexibility, particularly in the surface regions, suggesting a less rigid but still functionally relevant binding interaction.

These findings suggest that ligand binding plays a significant role in stabilizing the protein’s conformation, particularly in the binding site, which could have functional implications for the protein’s activity and interactions with other biomolecules.

## RMSF

**Fig. 6:**
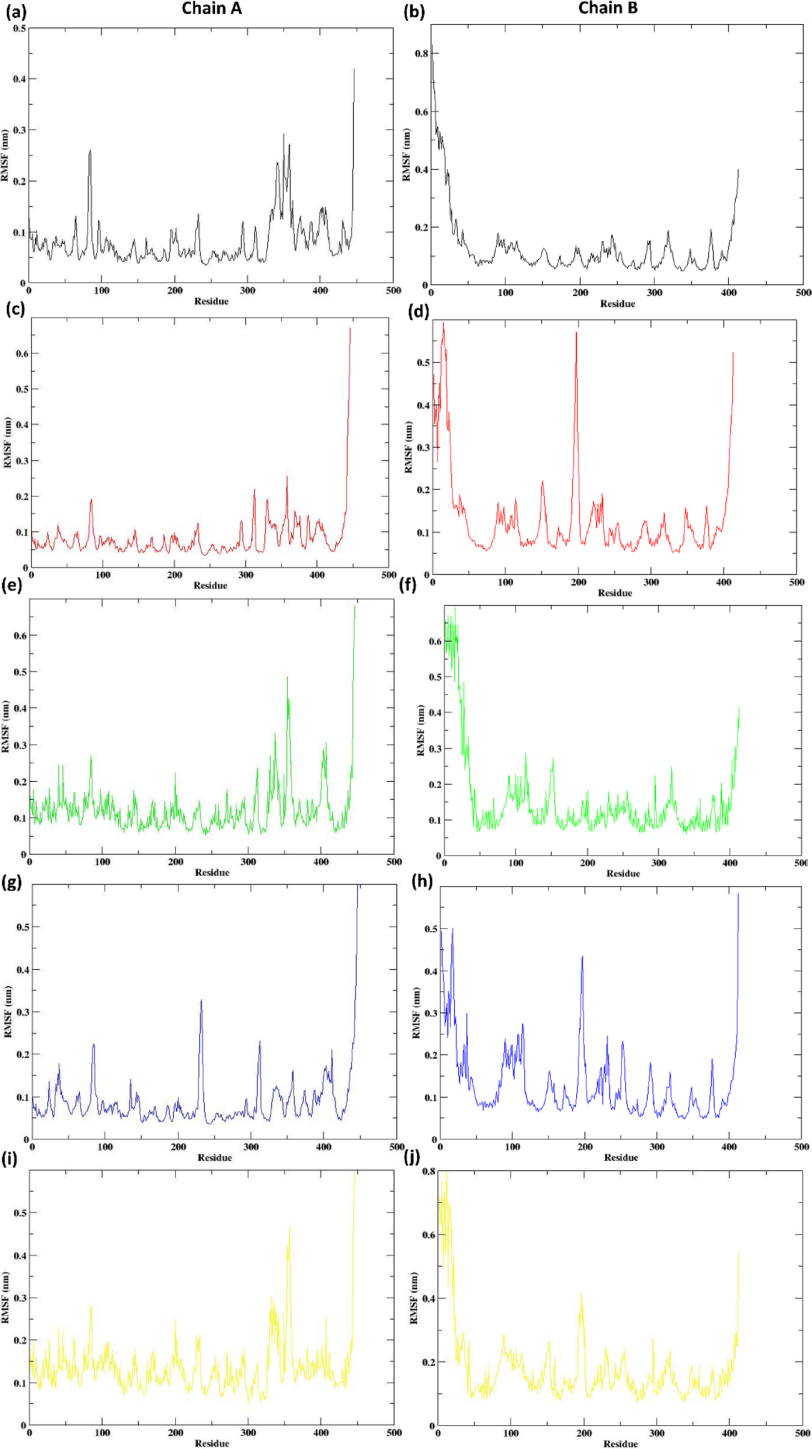
RMSF of protein Cα atoms when bound to the ligands in Chain A Chain B for the (**a**, **b**) Apo protein (**c**, **d**) Control (**e**, **f**) 638024 (**g**, **h**) 122623 (**i**, **j**) 197835.

The RMSF analysis was performed to evaluate the residue-wise flexibility of the Na⁺-NQR protein across the 300 ns simulation (Fig. 6). In general, the apo-protein exhibited higher fluctuations compared to the ligand-bound complexes, particularly in flexible loop regions and termini, indicating that ligand binding stabilized the protein structure.

In the apo system, significant fluctuations (RMSF > 0.3 nm) were observed at Chain A residues 10–24 and 104, and Chain B residues 84, 85, 350, 357, 369, and 407, which are mainly located within solvent-exposed loops. In contrast, ligand binding notably reduced these fluctuations, though some localized flexibility persisted.

In the control complex (Korormicin), elevated fluctuations were observed in Chain A at residues 3–6, 8, 10–11, 20–24, and 195, 199, 409, 410, and in Chain B at residues 442–445. The 638024 complex displayed localized flexibility in Chain B residues 3–19, indicating minor fluctuations at the N-terminal region. For 122623, higher fluctuations were noted at Chain B residues 8, 9, 11, 20, 22, and 196, 412, 413, as well as Chain A residues 84, 313, 444, and 445. The 197835 complex exhibited moderate fluctuations primarily in Chain A residues 354, 356, 357, 444, 445, and Chain B residues 197 and 412.

When comparing global trends across all systems, fluctuations remained largely below 0.3 nm for most of the protein backbone, indicating stable complex formation throughout the simulation. Among the complexes, 638024 demonstrated the most consistent RMSF profile, closely matching the control, suggesting a rigid and stable binding interaction. The complexes of 122623 and 197835 showed slightly elevated fluctuations, particularly at loop regions, but remained within acceptable ranges (generally < 0.7 nm), indicating stable binding with minor conformational adaptations. Overall, ligand binding improved structural stability relative to the apo form while allowing local flexibility in regions likely associated with ligand accommodation.

### B-factor (Atomic Fluctuation) analysis

Supplementary Figure S3 shows the comparison of B-factor (atomic fluctuations) of the Na⁺-NQR protein in both apo form and bound to the ligands: Control (Korormicin), **638024**, **122623**, and **197835**, for both Chain A (a) and Chain B (b). The B-factor values, which reflect the flexibility and rigidity of the protein backbone, indicate that higher B-factor values correspond to regions of the protein with greater atomic mobility, generally associated with more flexible regions.

In Chain A (Figure S3(a)), the apo protein (black line) exhibits significant fluctuations in B-factor values, particularly around residues 50–150, suggesting higher flexibility in these regions. Among the ligands, **122623** shows the lowest fluctuations, indicating that it stabilizes the protein structure most effectively. Both the Control (Korormicin) and **638024** complexes show intermediate B-factor values, with fluctuations observed in the flexible loop regions. **197835** shows the highest B-factor values, especially at residues 50–200, suggesting that it induces more conformational flexibility in the protein compared to the other ligands. For Chain B (Figure S3(b)), similar trends are observed. **122623** again shows the least fluctuation, implying that it promotes greater protein stability upon binding. The Control shows stable B-factors with some fluctuations in the loop regions, while **638024** and **197835** exhibit noticeable fluctuations, particularly in the terminal and loop regions, indicating that these compounds cause more conformational flexibility compared to **122623** and the Control.

Overall, the B-factor analysis reveals that ligand binding generally reduces the protein’s flexibility, stabilizing its structure. **122623** demonstrates the most stabilizing effect, while **197835** introduces greater conformational flexibility. The Control (Korormicin) and 638024 show intermediate effects, contributing moderate stabilization.

### Radius of gyration (Rg) analysis

**Fig. 7:**
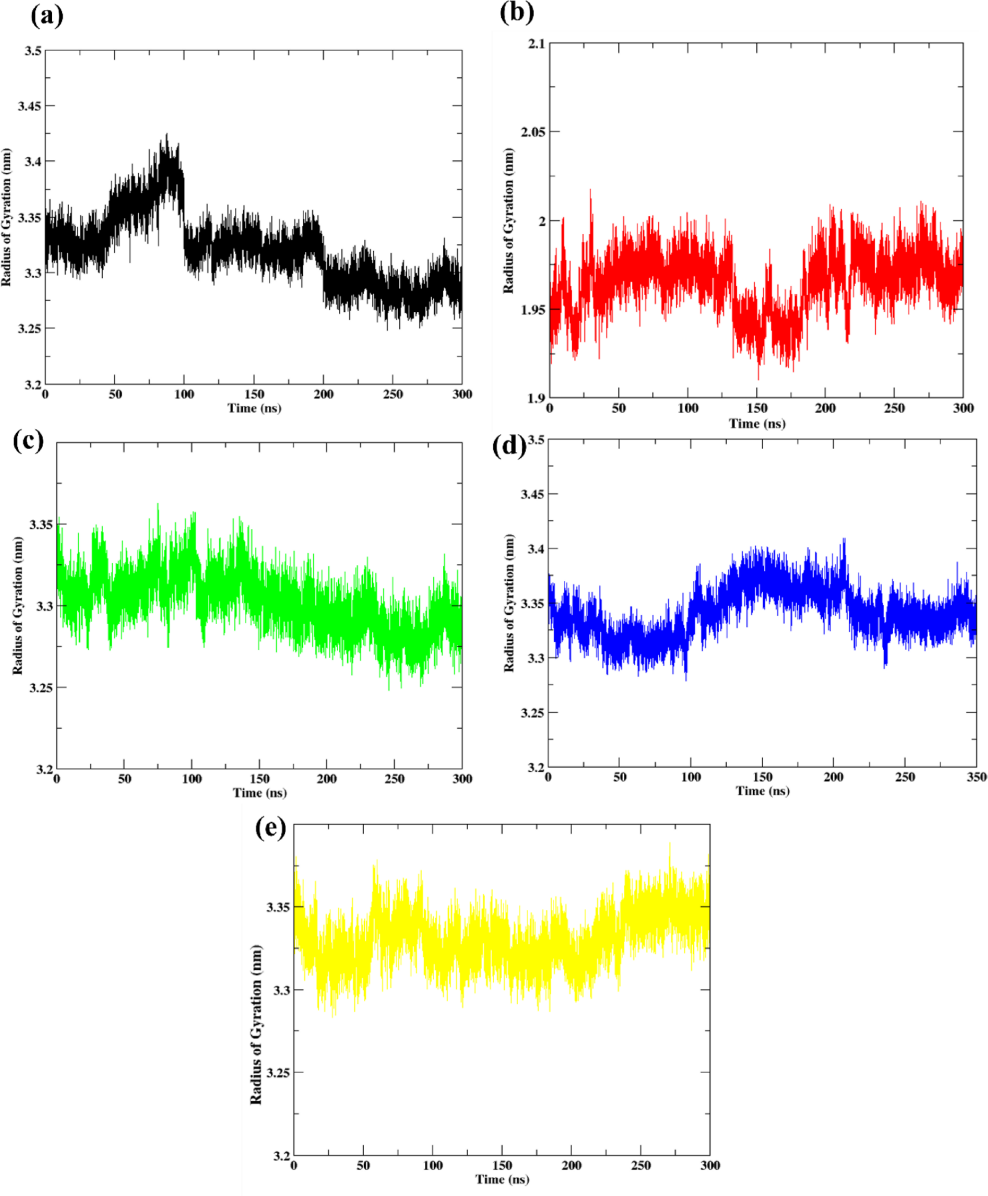
Radius of gyration for the protein for the (**a**) Apo protein (**b**) Control (**c**) 638024 (**d**) 122623 (**e**) 197835.

The radius of gyration (Rg) was evaluated to assess the compactness and global conformational stability of the Na⁺-NQR protein in both apo and ligand-bound states throughout the 300 ns simulation (Fig. 7). For the apo protein (Fig. 7a), Rg fluctuated between 3.25 nm and 3.45 nm, with occasional larger deviations observed after ~ 100 ns. This indicates moderate flexibility and slight global conformational changes in the absence of ligand binding, consistent with prior RMSF results.

Upon ligand binding, a general reduction in Rg fluctuations was observed, indicating greater compactness and structural stability. Control (Korormicin) (Fig. 7b) maintained a highly stable Rg profile, fluctuating narrowly around 1.95 nm throughout the simulation, suggesting significant protein compaction and stabilization upon ligand binding. **638024** complex (Fig. 7c) showed Rg fluctuations in the range of 3.25–3.35 nm, which closely resembles the apo state but with reduced amplitude, indicating moderate compaction. **122623** complex (Fig. 7 d) exhibited slightly more fluctuation compared to **638024**, with Rg ranging between 3.30 and 3.40 nm, suggesting minor breathing motions but maintaining overall stability. **197835** complex (Fig. 7e) presented slightly higher Rg fluctuations around 3.25–3.35 nm, resembling the trend seen in the apo and **122623** systems, though with slightly more variation after 150 ns.

Overall, all ligand-bound complexes displayed improved compactness compared to the apo protein, suggesting that ligand binding stabilized the Na⁺-NQR structure. Among the ligands, Korormicin (control) showed the most compact and stable conformation, while **638024** and **197835** maintained acceptable structural integrity with minimal deviations.

#### Solvent accessible surface area (SASA) analysis

**Fig. 8:**
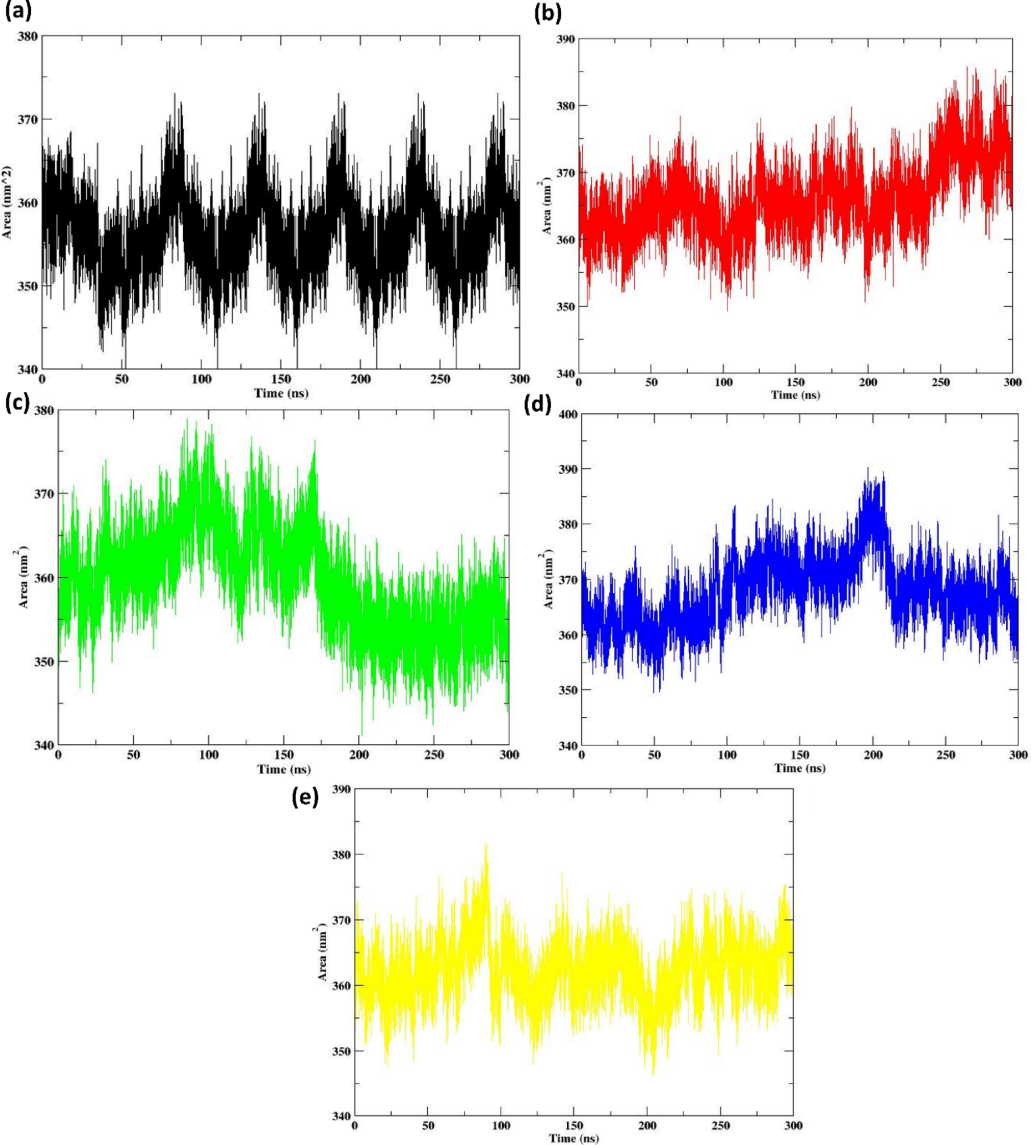
SASA for the protein for the (**a**) Apo protein (**b**) Control (**c**) 638024 (**d**) 122623 (**e**) 197835.

Figure 8 depicts the Solvent Accessible Surface Area (SASA) of the protein under the following conditions: (a) Apo protein, (b) Control, (c) 638024, (d) 122623, and (e) 197835, during a simulation duration of 300 ns. SASA is an essential feature that indicates the protein surface’s exposure to the solvent, offering insights on protein stability, conformational alterations, and ligand interactions. The Apo protein exhibits a variable SASA profile, characterised by cyclical augmentations and reductions in the accessible surface area. Such fluctuations are anticipated in an unbound protein, as it experiences dynamic structural alterations throughout the simulation. The overall trend seems constant, indicating no substantial structural changes or major conformational transitions during the simulation period. The Control protein demonstrates a more stable SASA in comparison to the Apo protein. Nonetheless, certain variants persist, suggesting that the protein structure has flexibility, albeit to a diminished degree. This consistency may suggest that the protein is in a comparatively stable state, exhibiting fewer structural variations. The 638024 compound-bound protein exhibits a significant increase in solvent-accessible surface area (SASA) compared to the Apo and Control proteins. This may result from the compound’s binding, potentially causing partial unfolding or alterations in the protein’s surface accessibility as it adjusts to accommodate the ligand. The observed variations in SASA may indicate a dynamic binding process, wherein the protein adapts to the compound’s attachment. The 122623 compound-bound protein exhibits a pattern of mild variations in SASA, akin to 638024, although demonstrates a more stable trajectory. This indicates that the binding of 122623 may elicit modest conformational alterations in the protein, resulting in a more stable exposure of the surface area relative to the protein associated with 638024. This may indicate a more stable binding relationship or reduced disruption of the protein structure.

#### Hydrogen bonds

**Fig. 9:**
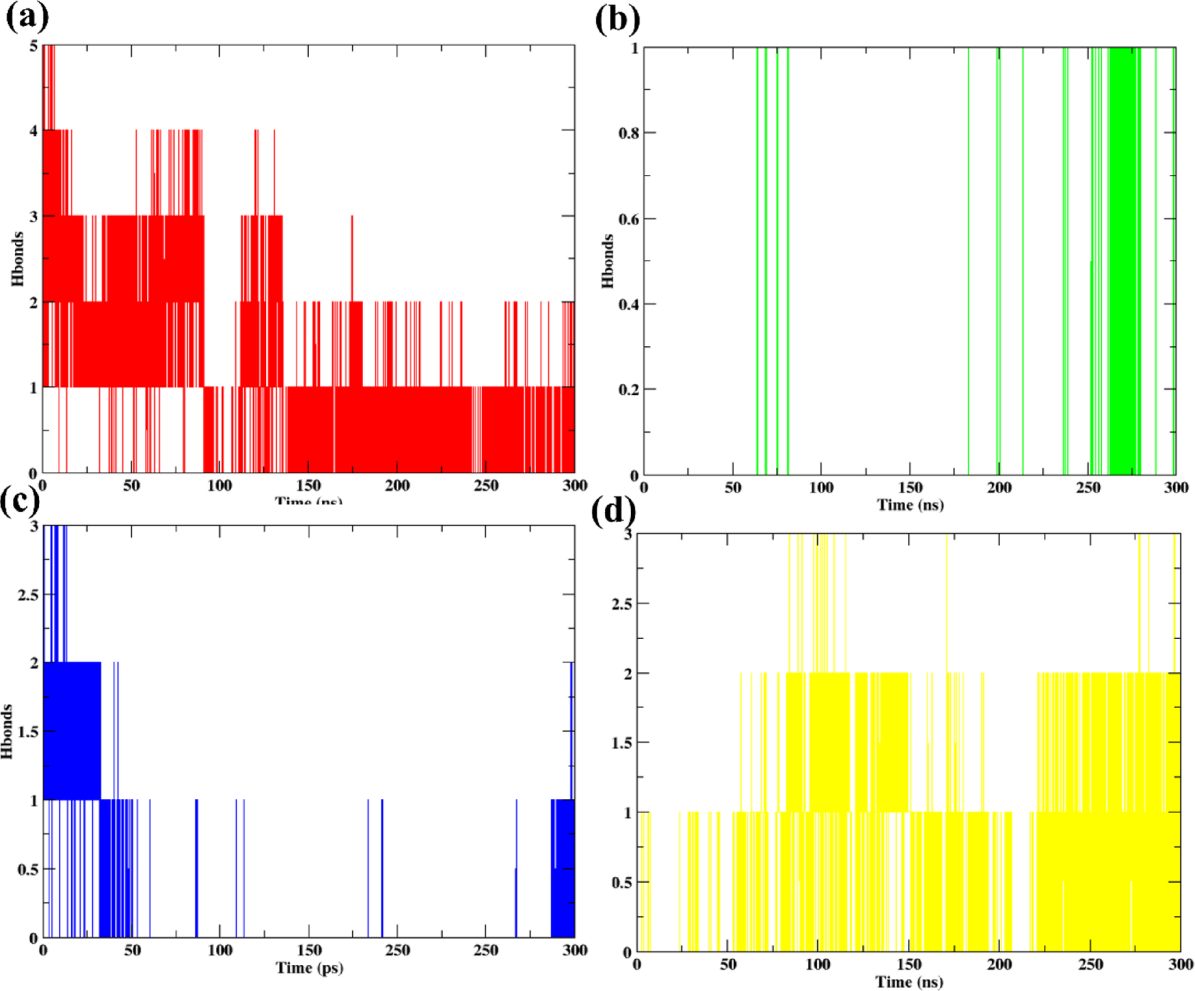
Hydrogen bonds formed between the protein ligand complex when bound to (**b**) Control (**c**) 638024 (**d**) 122623 (**e**) 197835 for 300 ns simulation.

The time-dependent hydrogen bond analysis between the protein and ligands over the 300 ns simulation is depicted in Fig. 9. The number and persistence of hydrogen bonds directly reflect the strength and stability of ligand binding.

Among all complexes, the control ligand Korormicin (Fig. 9a) exhibited the highest and most sustained hydrogen bond occupancy throughout the entire simulation. Multiple hydrogen bonds (ranging from 1 to as high as 5) were maintained for a considerable portion of the simulation time, indicating a highly stable and favorable binding interaction.

The **638024** complex (Fig. 9b) displayed limited hydrogen bond formation. Although hydrogen bonding events were sporadic, a few stable hydrogen bonds were observed primarily after 250 ns, suggesting late stabilization but overall weaker binding compared to the control. For **122623** (Fig. 9c), hydrogen bonds were observed mostly in the initial 50 ns, with the interaction becoming sparse after that. This intermittent bonding pattern indicates weaker or transient interactions with the binding site, similar to **638024** but with less favorable bonding over time. In the case of **197835** (Fig. 9 d), very few hydrogen bonds were formed throughout the simulation. Although short hydrogen bonding events occurred intermittently, no persistent hydrogen bonding network was established, suggesting a relatively weak interaction stability compared to the other ligands.

In summary, Korormicin exhibited the most stable hydrogen bonding profile, followed by **638024**, which showed occasional stable hydrogen bonds towards the later phase of the simulation. Both **122623** and **197835** demonstrated less favorable and more transient hydrogen bonding behaviors, with **122623** showing slightly stronger interactions later in the simulation than **197835**.

## Hydrogen bond occupancy analysis

The time-dependent hydrogen bond formation between the protein and ligands during the 300 ns simulation is shown in Fig. 8. Hydrogen bonding patterns were further quantified through hydrogen bond occupancy analysis (supplementary Table S3), providing a detailed view of interaction stability and key residues involved.

For the control ligand (Korormicin), multiple stable hydrogen bonds were observed throughout the simulation. The most persistent hydrogen bond was between SER233 and the ligand, with an occupancy of 20.91%, while ILE306 exhibited extremely high occupancy values across several binding modes (up to 33.58%), indicating strong and consistent stabilization. Additional interactions with residues such as THR236 (27.40%), ARG297 (2.54%), and ILE298 (up to 6.64%) further supported the stable binding profile.

In the case of **638024**, overall hydrogen bond occupancy was relatively lower, suggesting weaker or transient interactions. TYR257 displayed moderate occupancy at 0.81%, while other residues such as SER238 (0.35%) and ALA253 (0.02%) contributed limited interactions, indicating that the binding mode was less stabilized via hydrogen bonding.

The ligand **122623** showed intermediate hydrogen bonding behavior. MET305 demonstrated the highest occupancy of 10.69%, followed by LEU307 (8.18%) and ALA253 (3.70%). While fewer residues participated compared to the control, several of these interactions persisted for significant portions of the simulation, suggesting moderate stabilization.

For **197835**, hydrogen bond occupancy was highly concentrated on ASP230, which formed multiple interactions with the ligand, reaching an exceptional occupancy of 50.01%. Additional stabilization was contributed by HIS234, THR236, TYR257, and ILE298 with lower but significant occupancies (up to 5.35%). This indicates that although the number of hydrogen bonds fluctuated over time (Fig. 8c), a subset of residues consistently contributed to stable binding.

Overall, these occupancy results are consistent with the time-dependent hydrogen bonding plots, confirming that Korormicin formed the most stable and persistent hydrogen bonding network, while **122623** and **197835** established fewer but important hydrogen bonds contributing to moderate stability. **638024** exhibited the weakest hydrogen bond network among the selected hits.

## Principal component analysis

**Fig. 10:**
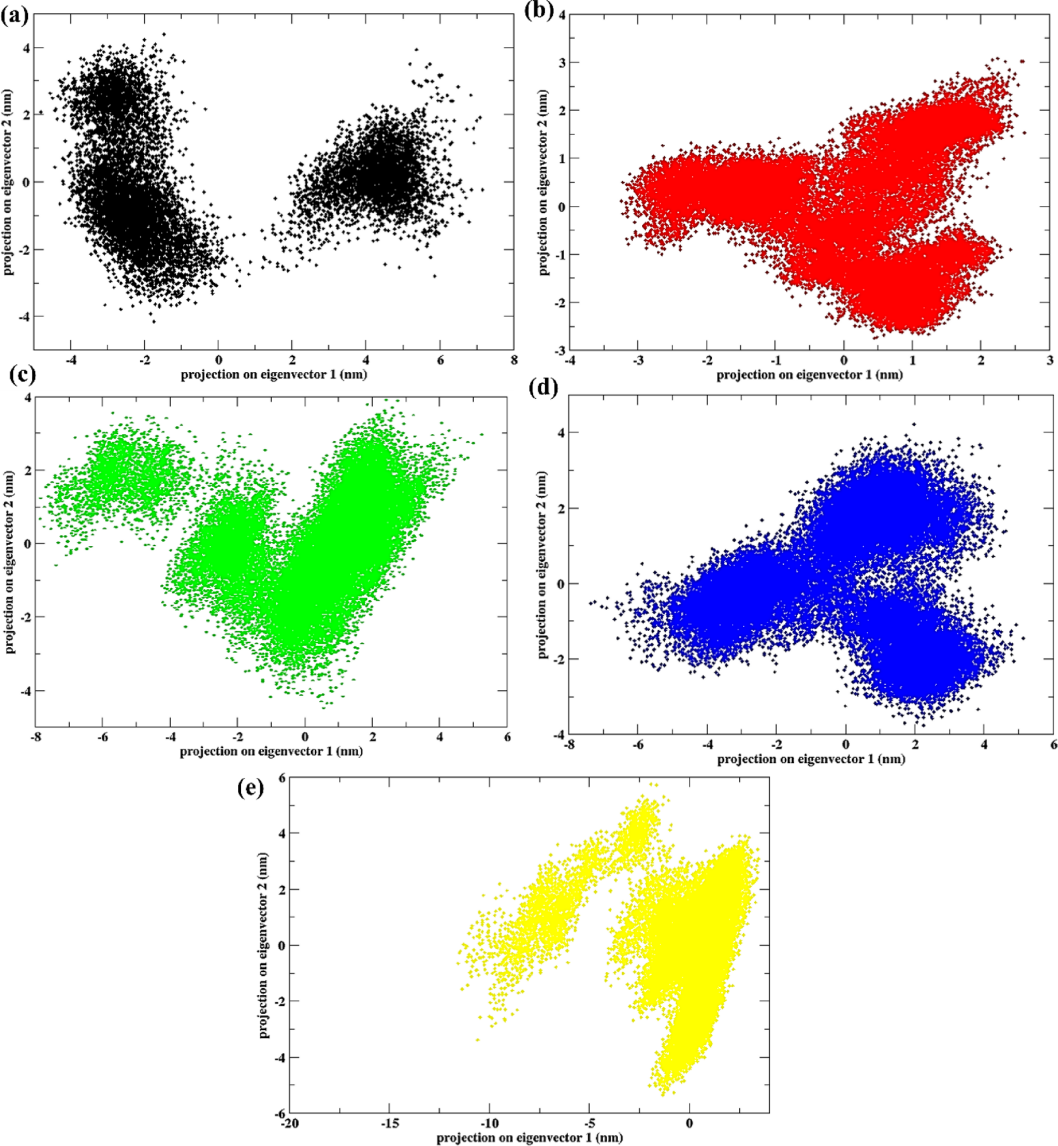
Principal component analysis for the protein-ligand complexes when bound to (**a**) Apo protein (**b**) Control (**c**) 638024 (**d**) 122623 (**e**) 197835.

The scatter plots shown in Fig. 10 represent 2D projections of the trajectory from a principal component analysis (PCA) for protein-ligand complexes when bound to different molecules, including the control substance (Apo protein) and other compounds: Korormicin (b), **638024** (c), **122623** (d), and **197835** (e). PCA is a statistical method that helps retain trends and patterns while minimizing the complexity of high-dimensional data. The first two eigenvectors (PC1 and PC2) were found to capture over 70% of the total motion, with higher-order eigenvectors contributing progressively less to the overall variance, mainly representing minor local fluctuations.

In Fig. 10(a), the plot for the Apo protein shows a relatively dense and centralized distribution of data points, suggesting a stable and more confined conformational space. This indicates minimal dynamic fluctuation, which is characteristic of a stable, unbound state. In Fig. 10(b), the scatter plot for the control ligand, Korormicin, reveals a more distributed pattern, showing greater flexibility in the protein-ligand complex. This distribution reflects a broader conformational space, indicating that Korormicin induces some degree of flexibility in the complex, though still with a relatively stable behavior.

In Fig. 10(c), the compound **638024** demonstrates a more spread-out distribution of data points, implying a higher degree of conformational variability. The compound likely induces flexibility, with the protein-ligand complex exploring a wider range of conformations. The plot for **122623** in Fig. 10(d) shows a much denser cluster of points, which indicates a more confined and stable protein-ligand complex, suggesting that this compound stabilizes the complex, restricting the conformational space.

Finally, Fig. 10(e) for compound **197835** shows two distinct clusters of data points, suggesting the protein-ligand complex adopts two different conformations or states during the simulation. This bimodal distribution implies that the ligand induces a transition between conformations, highlighting its dynamic influence on the protein.

In summary, PCA provides valuable insights into the conformational dynamics of the protein-ligand complexes. The compact and centralized distribution observed for Apo protein indicates a stable, minimal motion state, while the more dispersed distributions observed for Korormicin, **638024**, and **197835** suggest increased conformational flexibility. Compound **122623** shows the highest level of stability, maintaining a more confined protein-ligand complex, while the other ligands induce varying degrees of conformational variability.

### Free energy landscape

**Fig. 11:**
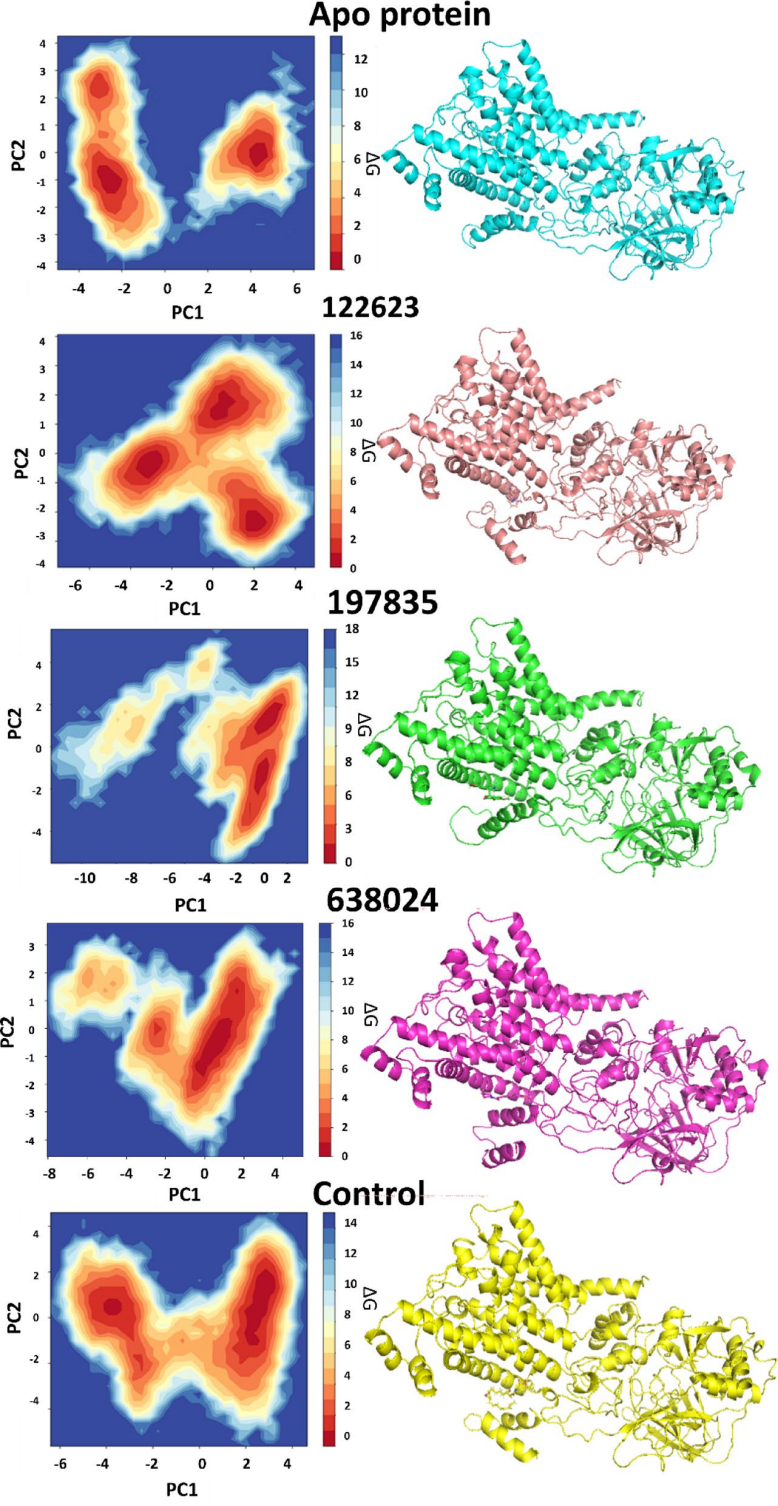
2D PCA-based FEL of the protein and their minimum energy structure in apo from and in complex with Control, 638024, 122623 and 197835.

Figure 11 illustrates the Free Energy Landscape (FEL) plots corresponding to a protein-ligand complex bound to various molecules: the control substance Korormicin, and the other compounds 122623, 197835, and 638024. FELs are commonly used in computational chemistry to represent the stability of different conformations within a molecular system.

In the plot for the apo protein (Fig. 11), a well-defined, deep basin is visible, indicating a stable conformational state for the protein without any bound ligand. The colour gradient, which indicates free energy, shows that the system is primarily in a stable state with lower energy regions marked in blue. The minimum energy structure for the apo protein is positioned in the deepest part of the basin, suggesting that this conformation is the most stable one in the absence of a ligand.

For the 122623 complex, the FEL reveals a less pronounced basin with higher free energy regions in yellow and orange. This suggests that the protein-ligand complex possesses a broader range of conformations with slightly higher energy, implying less stability compared to the control. The minimum energy structure for 122623 resides in a local energy well, indicating a stable conformation but with less defined energy minima compared to the apo or control states.

The 197835 complex presents a broad, shallow basin, indicating that the protein-ligand complex can adopt multiple conformations with similar free energy levels. The distribution of lower energy states (blue regions) implies a higher degree of flexibility and multiple potential stable states for the complex, with the minimum energy structure representing one of these flexible conformations.

For 638024, multiple shallow basins are observed, reflecting the presence of several stable or semi-stable conformations with comparable free energy. The intricate topography of the landscape suggests that the protein-ligand complex experiences variations among several conformational states, indicating less tendency to stabilize in a single conformation. The minimum energy structure in this case is representative of a conformation that is one of several possible low-energy states.

The control ligand, Korormicin, maintains the protein-ligand complex in a highly stable state, as evidenced by the narrow, deep basin observed in the FEL. The minimum energy structure for Korormicin is the most defined and stable, residing in the lowest part of the basin. In contrast, the other compounds (122623, 197835, and 638024) lead to more complex landscapes with broader, shallower basins, suggesting that these compounds induce multiple stable or semi-stable states. These findings indicate that each ligand interacts with the protein in a unique way, influencing the protein’s conformational dynamics and overall stability, which could be functionally significant.

#### MM/GBSA

The MM/GBSA results presented in Table 7 provide insight into the binding affinities of the protein-ligand complexes with four different ligands: the control substance (Korormicin), and the compounds **122623**, **197835**, and **638024**. The control compound, Korormicin, demonstrates the most favorable binding, with a ΔG_TOTAL_ of −57.42 kcal/mol, indicating a stable and strong interaction with the protein. This is supported by favorable van der Waals interactions (ΔE_VDWAALS_ = −65.29 kcal/mol) and electrostatic interactions (ΔEEL = −4.96 kcal/mol), which contribute to the overall stability. However, the ΔE_GB_ value of 21.67 kcal/mol and ΔE_SURF_ of −8.83 kcal/mol suggest that the solvation effects, particularly electrostatic solvation, play a significant role in stabilizing the protein-ligand complex.

In comparison, **638024** shows a significantly weaker binding affinity with a ΔG_TOTAL_ of −24.13 kcal/mol. While the van der Waals interactions (ΔE_VDWAALS_ = −35.14 kcal/mol) are still favorable, the electrostatic interactions (ΔEEL = −6.94 kcal/mol) are less pronounced, and the electrostatic solvation energy (ΔE_GB_ = 22.74 kcal/mol) is less stabilizing than the control. Additionally, the ΔG_SOLV_ value of 17.95 kcal/mol suggests that the solvation effect is less favorable, leading to a less stable overall complex.

The binding affinity of **122623** is intermediate, with a ΔG_TOTAL_ of −26.26 kcal/mol, showing that the van der Waals interactions (ΔE_VDWAALS_ = −38.71 kcal/mol) and electrostatic interactions (ΔE_EL_ = −9.88 kcal/mol) contribute positively to the binding. However, the electrostatic solvation energy (ΔE_GB_ = 27.5 kcal/mol) is higher than that of the control, and the ΔG_SOLV_ value of 22.33 kcal/mol indicates that the solvation effect is not as stabilizing as for Korormicin, weakening the overall binding.

Finally, **197835** has the weakest binding affinity, with a ΔG_TOTAL_ of −22.37 kcal/mol. Although the van der Waals interactions (ΔE_VDWAALS_ = −30.68 kcal/mol) and electrostatic interactions (ΔE_EL_ = −15.56 kcal/mol) are favorable, the electrostatic solvation energy (ΔE_GB_ = 28.29 kcal/mol) is the highest among the compounds, suggesting that solvation effects are less favorable for this complex. The ΔG_SOLV_ of 23.87 kcal/mol indicates that the ligand is less stabilized by solvation, contributing to the weaker overall binding affinity.

In conclusion, the MMGBSA results reveal that Korormicin maintains the most stable binding with the protein, with the other compounds (**122623**, **197835**, and **638024**) showing varying degrees of weaker stability, primarily due to less favorable solvation energies. The differences in the free energy components reflect how each ligand interacts with the protein and the solvent, influencing the overall stability of the protein-ligand complex.

**Table 7:** MMGBSA of the protein ligand complex when bound to control (Korormicin), 122623, 197835, 638024.

Compounds	ΔVDWAALS (kcal/mol)	ΔEEL (kcal/mol)	ΔEGB (kcal/mol)	ΔESURF (kcal/mol)	ΔGGAS (kcal/mol)	ΔGSOLV (kcal/mol)	ΔTOTAL (kcal/mol)
Control	−65.29	−4.96	21.67	−8.83	−70.26	12.84	−57.42
638024	−35.14	−6.94	22.74	−4.79	−42.08	17.95	−24.13
122623	−38.71	−9.88	27.5	−5.17	−48.59	22.33	−26.26
197835	−30.68	−15.56	28.29	−4.42	−46.24	23.87	−22.37

### Cross-validation of docking, MMGBSA, and ML-based binding predictions

A comparative analysis integrating molecular docking, MMGBSA binding free energy, and PSICHIC-predicted pKd values demonstrated overall consistency in ranking. Compound **197835** exhibited the strongest binding in both molecular docking (− 9.6 kcal/mol) and PSICHIC-predicted affinity (pKd 4.81). MMGBSA analysis revealed that compound **122623** displayed slightly more favorable binding free energy (− 25.03 kcal/mol) compared to **197835** (− 24.15 kcal/mol) and **638024** (− 24.45 kcal/mol), indicating potentially better stabilization post-complex formation. The control compound Korormicin showed moderate docking score (− 8.0 kcal/mol), high PSICHIC-predicted pKd (4.79), and significantly stronger MM/GBSA ΔG (− 48.73 kcal/mol), suggesting pronounced stabilization under dynamic conditions. The consistent trends across methodologies collectively support both **197835** and **122623** as promising candidates.

#### Per-residue decomposition

**Fig. 12:**
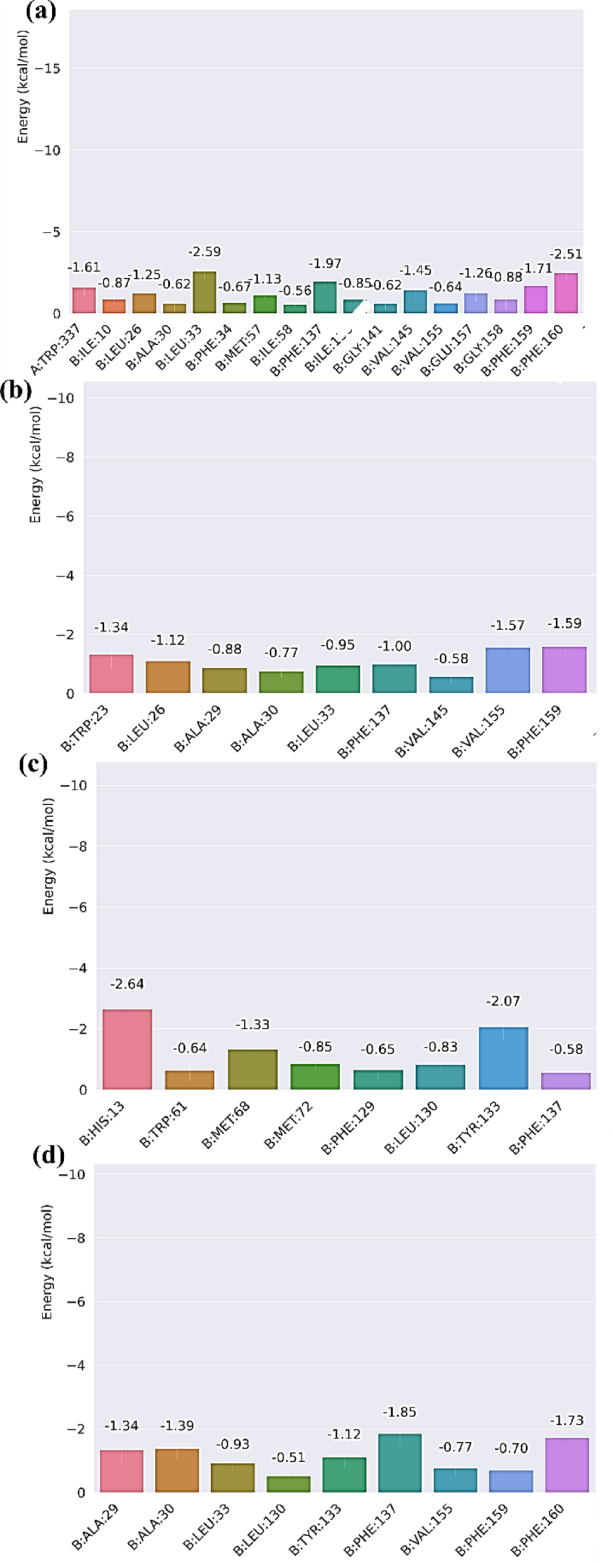
Per-residue decomposition of the protein-ligand complex when bound to (**a**) Control (Korormicin) (**b**) 122623 (**c**) 197835 (**d**) 638024.

The Fig. 12 represents bar graphs depicting the per-residue breakdown of the binding free energy for a protein-ligand complex when bound to different ligands: the control (Korormicin), and compounds **122623**, **197835**, and **638024**. Per-residue decomposition is a common analysis in computational drug design, which sheds light on the manner in which particular amino acid residues contribute to the overall association between ligands and proteins. The contribution made by individual residues is depicted in the graphs using bars with the size of the bar showing the extent of contribution that a particular residue makes on the binding free energy. Negative values generally indicate favourable contributions to binding affinity.

The energy contributions from residues when the protein is bound to Korormicin are quite dispersed, with several residues contributing significantly to binding. Specifically, the residues Leu33, Phe160, Phe137, Phe159, Val145, Glu157, Met57, Leu26 of chain B and Trp337 of chain A. This suggests that multiple interactions are stabilizing the ligand-protein complex, which is characteristic of a tight and specific binding. The plot for **122623** shows less residues with significant aids to the binding energy equated to the control. This might imply that while **122623** forms a stable complex with the protein, it may not interact with as many residues as Korormicin does or the interactions are not as energetically favorable. However, most of the interaction residues such as Phe159, Val155, Val145, Phe137, Leu33, Ala30, Leu26 are common with the interaction residues of the control. The contributions to binding energy from residues with **197835** bound are even less than with 122623, and the overall magnitude of the contributions is lower. Only a single residue Phe137 was found to be common from the interaction residues of the control. This could indicate weaker or fewer interactions between the ligand and protein, which may correlate with a lower binding affinity. Similar to **122623**, **638024** shows limited and lower magnitude contributions across the residues, however it showed multiple residues Leu33, Phe160, Phe137, Phe159, Ala30 and Val155 which were common interacting residues for the control.

The per-residue energy decomposition helps in identifying key residues that contribute to the binding affinity. Residues with higher negative contributions are often crucial for binding and can provide targets for enhancing ligand interactions to improve drug efficacy. The control compound, Korormicin, appears to have a more distributed and stronger interaction across multiple residues, which is often associated with more specific and stronger binding. However, both **122623** and **638024** also showed the next best interaction with most residues.

## Triplicates MD simulation

The supplementary figure S4 displays triplicates of the 300 ns RMSD simulation for both the protein and ligand across different conditions: Apo protein, Control, **638024**, **122623**, and **197835**. The RMSD of the protein reflects its structural stability over time, while the ligand RMSD highlights its binding stability. For the protein RMSD, the Apo protein (a) exhibits stable fluctuations after an initial adjustment, indicating that the protein adopts a stable conformation, with minimal structural deviations. The Control protein (b) shows similarly stable behavior, although with slightly higher fluctuations, possibly due to minor structural adjustments induced by ligand binding. The conditions with ligands **638024** (d), **122623** (f), and **197835** (h) display larger RMSD fluctuations, suggesting that these ligands induce greater conformational flexibility in the protein. This increased flexibility could be due to altered interactions between the protein and ligand, leading to more dynamic changes in the protein’s structure during the simulation.

For the ligand RMSD, the Control condition (c) shows minimal fluctuations, suggesting that the ligand remains stably bound to the protein’s active site throughout the simulation. In contrast, ligands **638024** (e), **122623** (g), and **197835** (i) exhibit higher RMSD fluctuations, indicating less stable binding or transient detachment events from the active site. The higher RMSD values for these ligands suggest that they are less tightly bound to the protein, which may result in weaker interactions and possibly less favorable binding affinity. Overall, the Apo and Control conditions show more stability in both protein and ligand RMSD, while the ligands **638024**, **122623**, and **197835** induce more dynamic behavior, potentially implying weaker or less stable binding interactions. This analysis suggests that the binding of these ligands affects the structural stability of the protein and ligand, with **638024**, **122623**, and **197835** leading to more significant conformational changes compared to the Control.

## Network Pharmacology

**Fig. 13:**
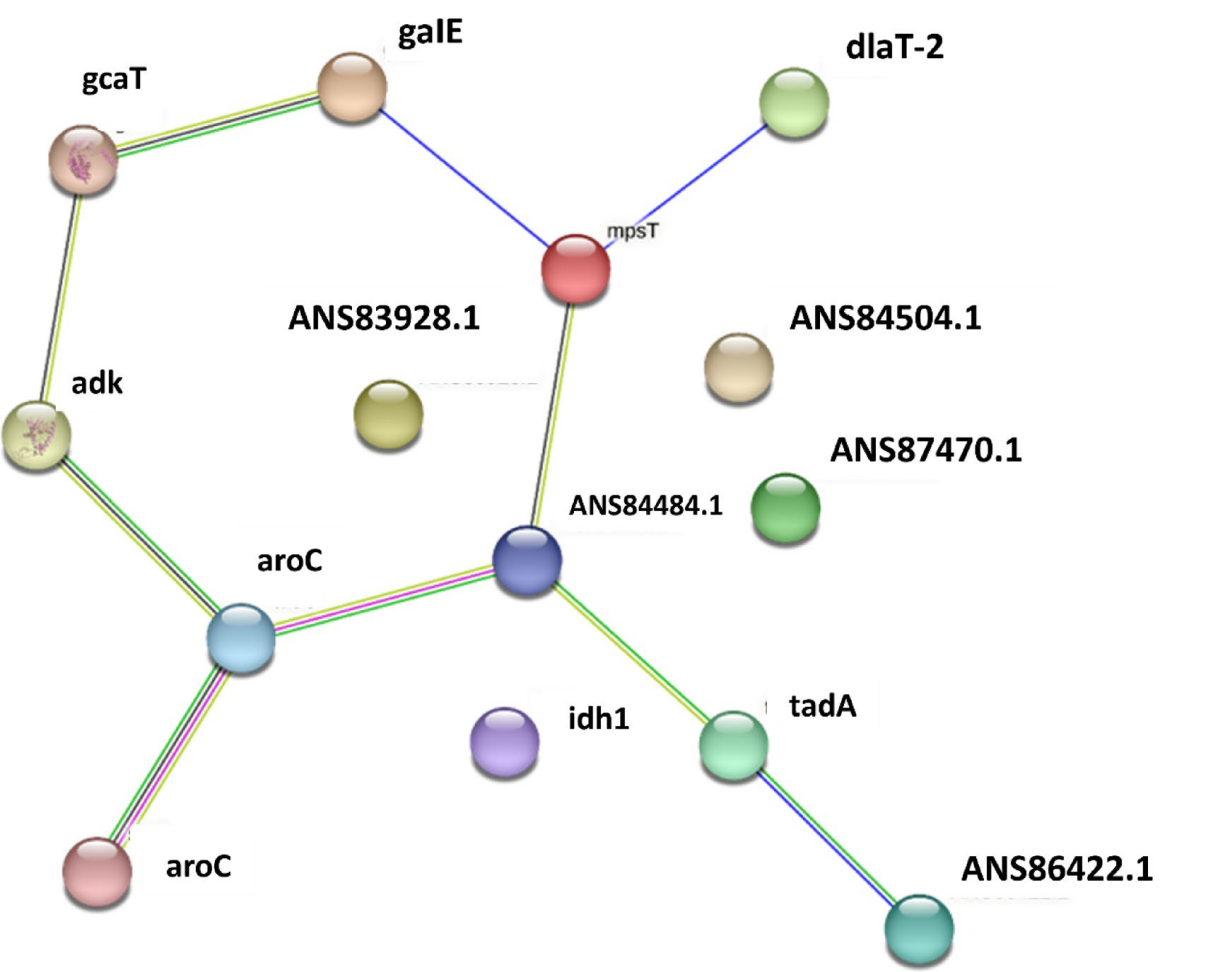
Protien – protein interaction network of the predicted protein constructed by STRING databse. The different color codes represented each compound and interaction.

Although cholera is primarily an infectious disease caused by *Vibrio cholerae*, network pharmacology was employed in this study to explore potential host-related interactions and systemic effects of the selected phytochemicals. DIGEP-prep web server was used to analyze the networking of the selected chemical compounds. In this approach, we were able to determine that the following are the genes linked with the protein that is attacked by each compound. Moreover, the target set comprising the genes predicted to be down-regulated by these compounds was specified. The target genes for compound **197835**, **122623**, and **638024** were obtained in accordance with these predictions: 500, 27, and 500, respectively. Finally, 1027 genes were obtained and used for uploading to the STRING database to predict the protein-protein interaction as shown in Fig. 13.

**Fig. 14:**
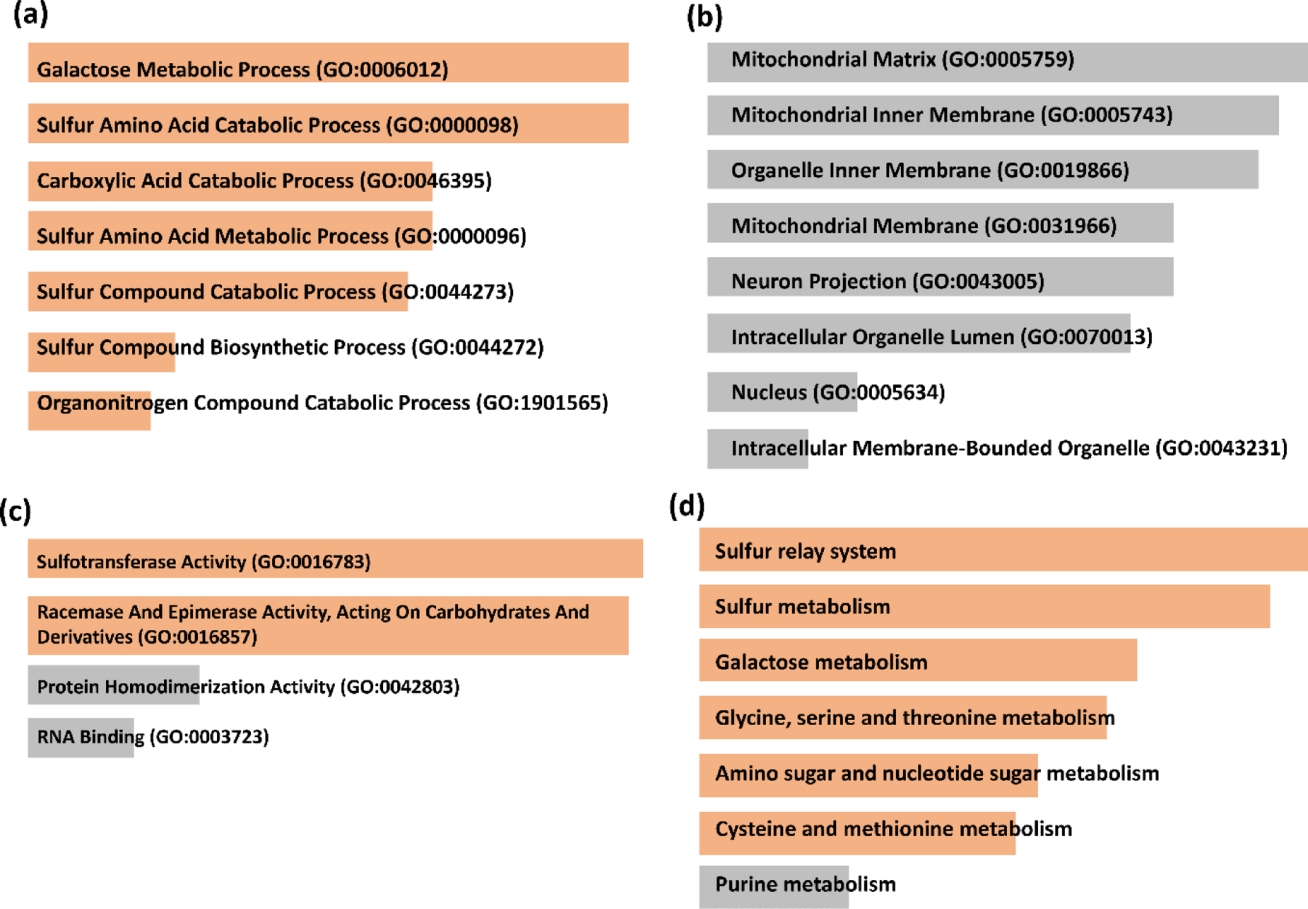
Illustrating the Enrichr analysis of predicted genes from the string database.

**Fig. 15:**
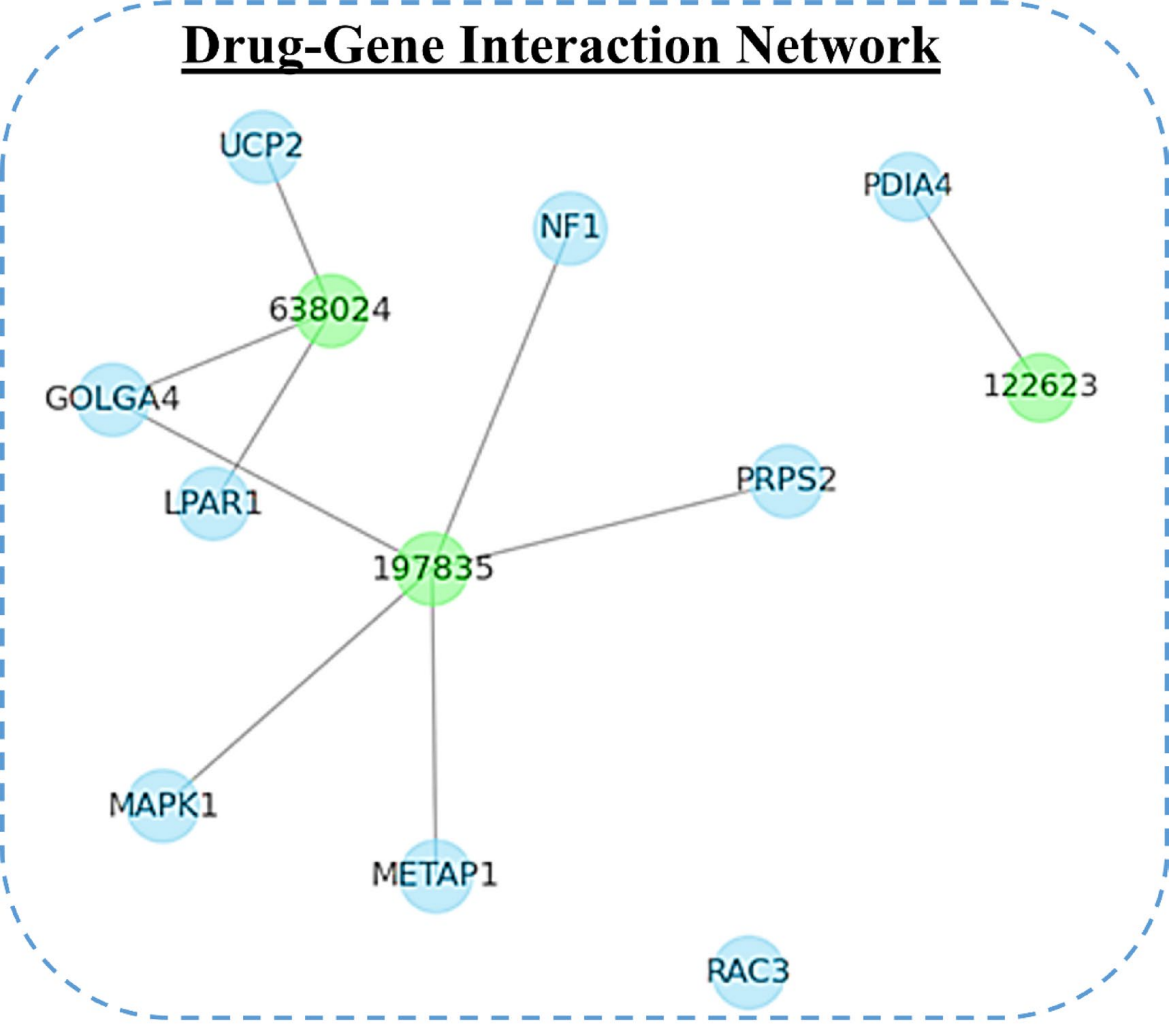
Drug-protein interaction analysis of predicted genes.

In addition, employing the Enirchr webserver the gene sets were classified according to their biological process. According to Gene Ontology analysis, target genes are involved in various cellular processes, especially in catabolic and metabolic processes. Furthermore, they are involved in molecular activities related to sulfurtransferase activity and RNA binding. Moreover, the interaction networks of their cellular components were also decoded. Gene ontology analysis of these genes revealed that they are mostly involved in sulfur and carbohydrate metabolism based on the KEGG pathway (Fig. 14). Additionally, gene-drug interaction profiling suggested that the compounds may have therapeutic relevance beyond cholera, potentially affecting diseases such as *Vibrio scophthalmi* infection, thereby broadening their possible applications (Fig. 15). This network pharmacology approach provided a systems-level perspective on the multi-target potential, safety, and versatility of the screened phytochemicals. This led to further network analysis to indicate that these compounds might also be efficient against diseases like *Vibrio scophthalmi* which could make them more versatile in medical applications.

## Conclusion

The computational analyses conducted in this study identified promising compounds from *Berberis vulgaris* and *Hydrastis canadensis* that may serve as effective anti-cholera agents. The compounds exhibited strong binding affinities to the cholera toxin, with several showing comparable or superior profiles to the control compounds. Among these, the top three compounds 122623, 197835, and 638024 were found to have better binding scores than the control. The integration of DFT, ADMET, and interaction property analysis with ML-based binding affinity prediction has established a comprehensive framework for the identification of potential anti-cholera compounds. RMSD of the ligands showed that all three were stable in the binding pocket. MM/GBSA showed that **122623** had better binding free energy than **197835** and **638024**. The per-residue energy decomposition highlighted critical interactions at the molecular level that contribute to the binding efficiency of these compounds. These findings suggest that *Berberis vulgaris* and *Hydrastis canadensis* are valuable sources for natural anti-cholera agents and warrant further investigation. While the current study provides in silico evidence for potential Na⁺-NQR inhibitors, experimental validation through in vitro binding and functional assays remains essential and is planned for future investigation. The use of computational drug discovery tools accelerates the identification process, paving the way for subsequent in vitro and in vivo validation. If future lab studies confirm these results, the compounds could help develop new plant-based treatments for cholera. This may provide practical solutions for enhancing patient outcomes, particularly in areas where current therapies are limited.

## Supplementary Information

Below is the link to the electronic supplementary material.

Supplementary Material 1

## Data Availability

The datasets generated and/or analysed during the current study are available upon request from the corresponding author.
