# Supplementary material for: Machine learning-guided in Silico identification of Na⁺-NQR inhibitors from Berberis vulgaris and Hydrastis Canadensis phytochemicals against Vibrio cholerae
Source: Sci Rep. 2025 Nov 12;15:39726. doi: 10.1038/s41598-025-23546-2 (PMC12612261; doi:10.1038/s41598-025-23546-2)
Supplement: Supplementary file 1 — Supplementary Material 1 [file 41598_2025_23546_MOESM1_ESM.docx]

**Supplementary**

**Table S1.** Phytochemicals of *Hydrastis canadensis* and *Berberis vulgaris* found from Natural Product Activity and Species Source Database (NPASS)

| *Hydrastis canadensis* | |
| --- | --- |
| Natural Product ID | **SMILES** |
| NPC296482 | COc1c(OC)ccc2c1c[n+]1CCc3c(c1c2)cc1c(c3)OCO1.[Cl-] |
| NPC136330 | O=C(N1CCCCC1)/C=C/C=C/c1ccc2c(c1)OCO2 |
| NPC53069 | COc1ccc2cc3-c4cc5c(cc4CC[n+]3cc2c1OC)OCO5 |
| NPC27887 | O=Cc1c(ccc(c1OC)OC)CC1N(C)CCc2c1cc1OCOc1c2 |
| NPC216459 | COc1c(OC)ccc2c1CN1CCc3c([C@@H]1C2)cc1c(c3)OCO1 |
| NPC138487 | COc1ccc2C[C@@H]3c4cc5c(cc4CCN3Cc2c1OC)OCO5 |
| NPC73020 | CN1CCc2cc3c(cc2[C@@H]1[C@@H]1c2ccc(c(c2C(=O)O1)OC)OC)OCO3 |
| NPC159275 | COc1c(C)c2oc(cc(=O)c2c(c1C)O)c1ccc(cc1)O |
| NPC54379 | COc1cc2CCN3[C@H](c2cc1O)Cc1c(C3)c(OC)c(cc1)OC |
| NPC78913 | COc1cc2oc(cc(=O)c2c(c1C)O)c1ccc(cc1)O |
| NPC14622 | CN1CCc2cc3c(cc2C1[C@@H]1c2ccc(c(c2C(=O)O1)OC)OC)OCO3 |
| NPC477259 | COC1=C(C2=C(CC3C4=CC5=C(C=C4CCN3C2=O)OCO5)C=C1)OC |
| NPC482982 | Cc1c(c(C)c2c(c(=O)c(c(c3ccc(c(c3)O)OC)o2)O)c1OC)OC |
| NPC24228 | COc1c(ccc2CC3c4cc5c(cc4CCN3C(=O)c12)OCO5)O |
| NPC477258 | CC(=O)OC1=C(C2=C(CC3C4=CC5=C(C=C4CCN3C2=O)OCO5)C=C1)OC |
| NPC167084 | Cc1c(cc(c2c(=O)cc(c3ccc(cc3)O)oc12)O)OC |
| NPC478985 | COc1cc(ccc1O[C@H]1[C@@H]([C@H]([C@@H]([C@@H](CO)O1)O)O)O)/C=C/C(=O)O[C@@H]1C[C@@](C[C@H]([C@H]1O)O)(C(=O)O)O |
| NPC170826 | CC[C@H](CC[C@@H](C)[C@H]1CC[C@H]2[C@@H]3CC=C4C[C@H](CC[C@]4(C)[C@H]3CC[C@]12C)O[C@@H]1[C@@H]([C@H]([C@@H]([C@@H](CO)O1)O)O)O)C(C)C |
| NPC269699 | COc1ccc2cc3-c4cc5c(cc4C(C[n+]3cc2c1OC)O)OCO5 |
| NPC93989 | CN1CCc2cc3c(cc2[C@H]1Cc1ccc(c(c1C=O)OC)OC)OCO3 |
| NPC183485 | COc1ccc2cc3-c4cc5c(cc4CC[n+]3cc2c1OC)OC(O5)[3H] |
| NPC122483 | Cc1c(c2c(=O)cc(c3ccc(c(c3)O)O)oc2c(C)c1OC)O |
| NPC308036 | c1cc2c(c[nH]c2cc1Br)CSCCN |
|  | ***Berberis vulgaris*** |
| NPC78359 | COc1ccc2c3c1O[C@@H]1[C@@]3(CCN(C2)C)C=C[C@@H](C1)O |
| NPC53069 | COc1ccc2cc3-c4cc5c(cc4CC[n+]3cc2c1OC)OCO5 |
| NPC7715 | COc1cc2CCN([C@H]3c2cc1Oc1c2c(CCN([C@H]2Cc2ccc(Oc4cc(C3)ccc4O)cc2)C)cc(c1O)OC)C |
| NPC47298 | C/C=C/1[C@H]2C=C(C)C[C@@]1(c1ccc(nc1C2)O)N |
| NPC4669 | COC1=CC=c2c(C1=O)c[n+]1c(c2)c2cc3OCOc3cc2CC1 |
| NPC49075 | CN1CCc2cc(c3cc2[C@@H]1Cc1ccc(cc1)Oc1cc(ccc1O)C[C@@H]1c2c(CCN1C)cc(c(c2O3)OC)OC)OC |
| NPC16452 | COc1ccc2cc3-c4cc(c(cc4CC[n+]3cc2c1OC)OC)OC |
| NPC181796 | CN1CCc2cc(c3cc2[C@@H]1Cc1ccc(cc1)Oc1cc(ccc1O)C[C@@H]1c2c(CCN1C)cc(c(c2O3)O)OC)OC |
| NPC480593 | c1ccc(cc1)COC(=O)N1CCC[C@H]1C(=O)N1CCC[C@H]1C=O |
| NPC116007 | COc1ccc2cc3-c4cc5c(cc4CCn3c(=O)c2c1OC)OCO5 |
| NPC251735 | CN1CCc2cc(c3cc2[C@@H]1Cc1ccc(c(c1)Oc1ccc(cc1)C[C@H]1c2c(CCN1C)cc(c(c2O3)OC)OC)O)OC |
| NPC480591 | CN1CCc2cc(c(c(c2C1)Oc1cc2c(CCN(C)C2Cc2ccc(cc2)O)cc1OC)OC)OC |
| NPC480590 | CCN(CC)Cc1cc2C[C@@H]3c4c(CCN3C)cc(c(c4Oc3cc4c(CCN(C)[C@H]4Cc4ccc(cc4)Oc(c2)c1O)cc3OC)OC)OC |
| NPC480592 | CN1CCc2cc(c(cc2C1Cc1ccc(c(c1)Oc1cc(ccc1OC)CC1c2cc(c(cc2CCN1C)OC)O)O)O)OC |
| NPC200388 | COc1cc(c2c(c1)oc(c1cc(c(cc1O)OC)O)c(c2=O)OC)O |
| NPC248109 | CN1CCc2cc(c3cc2C1Cc1cccc(c1)Oc1cc(ccc1O)CC1c2c(CCN1C)cc(c(c2O3)OC)OC)OC |
| NPC199402 | CN1CCc2cc(c3cc2[C@@H]1Cc1ccc(c(c1)Oc1ccc(cc1)C[C@@H]1c2c(CCN1C)cc(c(c2O3)OC)OC)O)OC |
| NPC117785 | CC(C)CCC[C@@H](C)CCC[C@@H](C)CCC[C@H](C)O |
| NPC300481 | CC(C)CCC[C@@H](C)CCC[C@@H](C)CCC[C@@H](C)O |
| NPC480589 | CN1CCc2cc(c(c(c2C1)Oc1cc2c(CCN(C)C2=O)cc1OC)OC)OC |


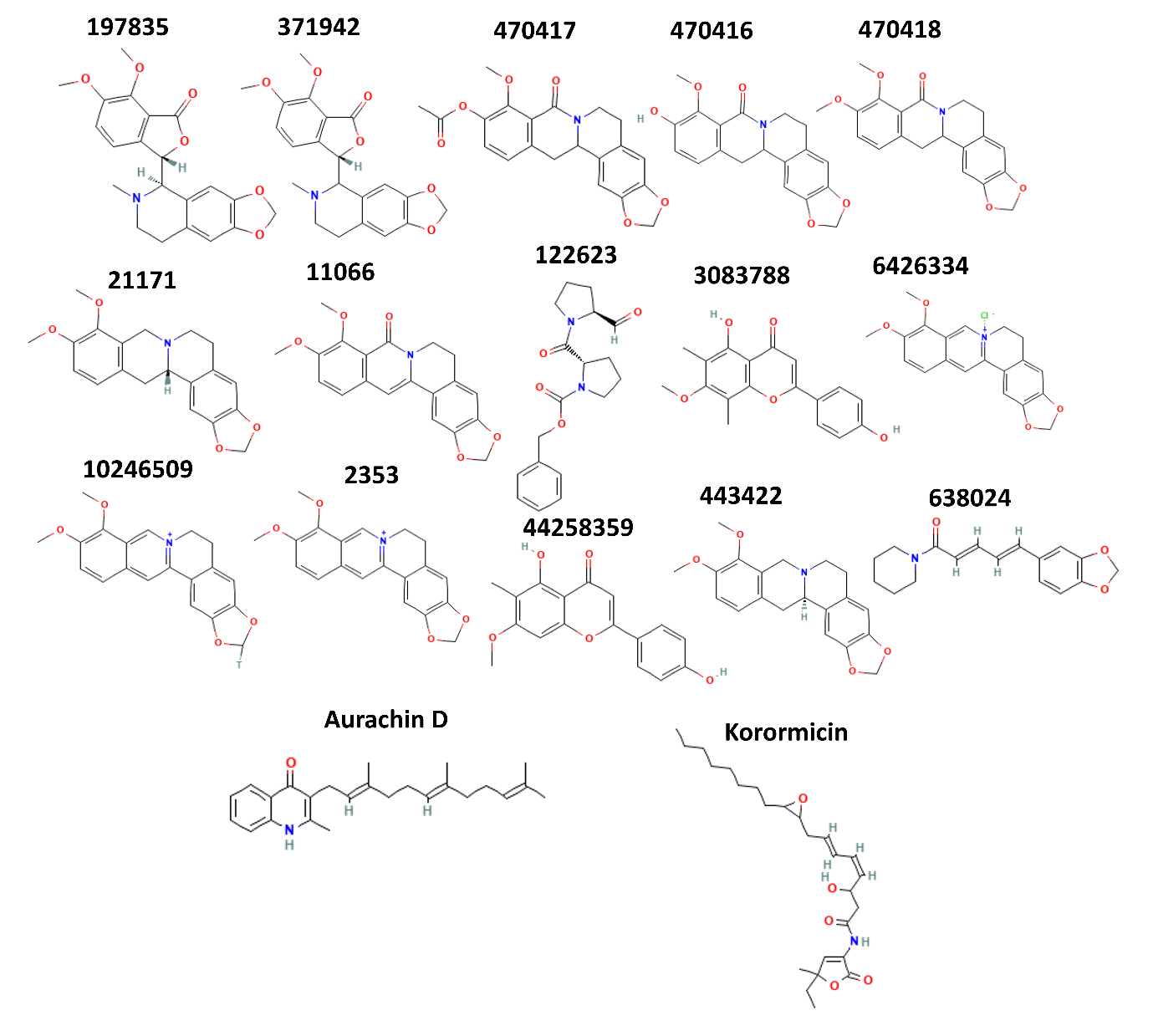


**Figure S1.** 2D representation of the to 15 compounds after virtual screening

**
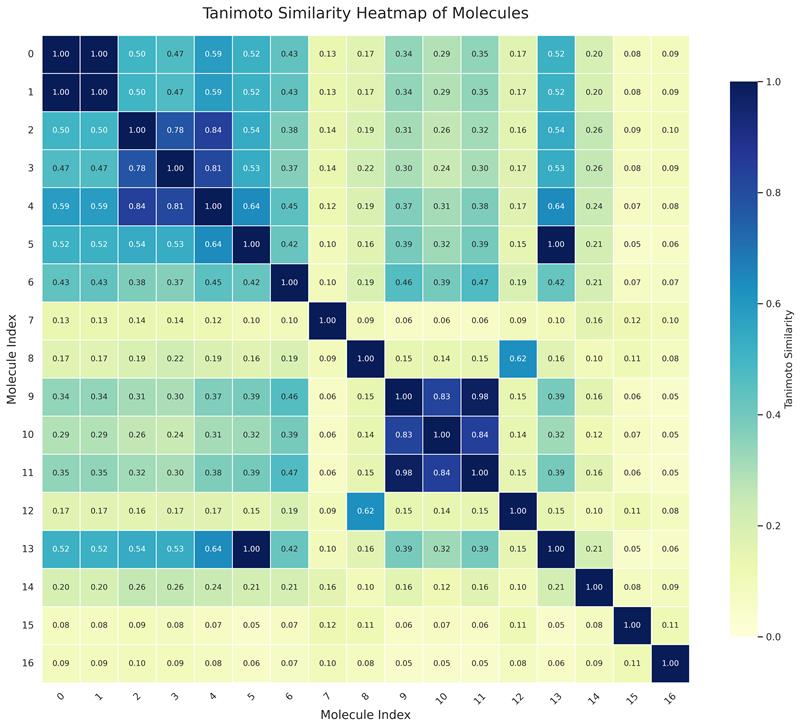
**

**Figure S2.** Tanimoto similarity of the to 15 compounds along with the two control compounds after virtual screening

**Table S2.** InChIKey analysis for the compounds 197835 and 371942

| Compound ID | InChI | InChIKey |
| --- | --- | --- |
| 197835 | InChI=1S/C21H21NO6/c1-22-7-6-11-8-15-16(27-10-26-15)9-13(11)18(22)19-12-4-5-14(24-2)20(25-3)17(12)21(23)28-19/h4-5,8-9,18-19H,6-7,10H2,1-3H3/t18-,19+/m1/s1 | JZUTXVTYJDCMDU-MOPGFXCFSA-N |
| 371942 | InChI=1S/C21H21NO6/c1-22-7-6-11-8-15-16(27-10-26-15)9-13(11)18(22)19-12-4-5-14(24-2)20(25-3)17(12)21(23)28-19/h4-5,8-9,18-19H,6-7,10H2,1-3H3/t18?,19-/m0/s1 | JZUTXVTYJDCMDU-GGYWPGCISA-N |


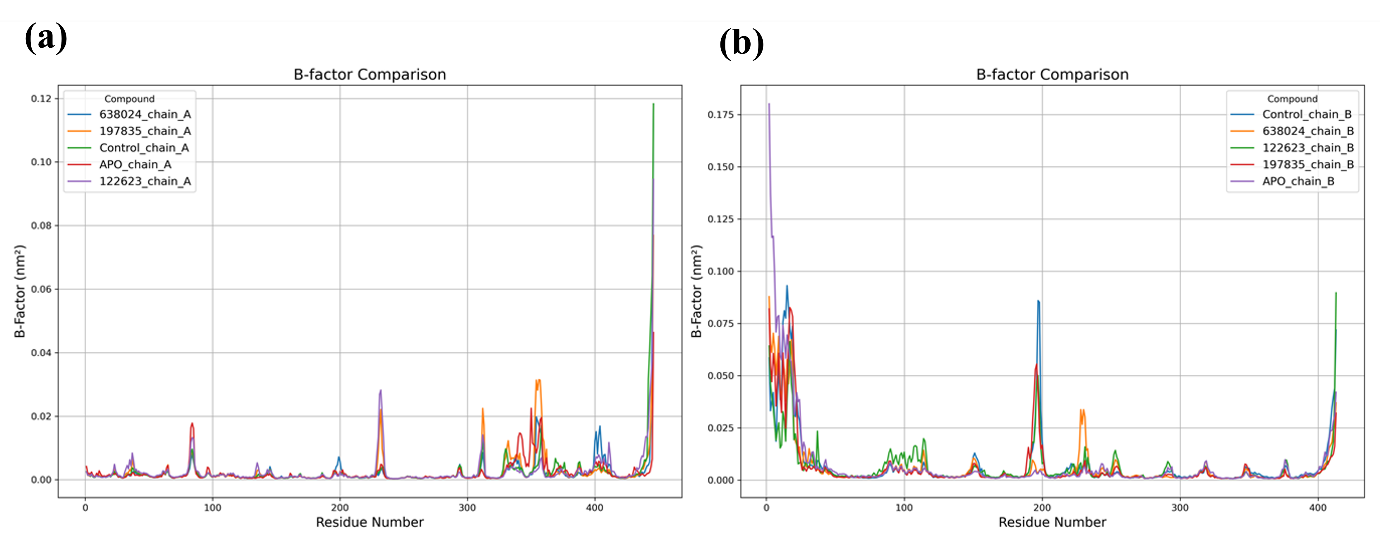


**Figure S3.** B-factor (atomic fluctuations) of the protein when in apo form and in bound state with Control, 638024, 122623 and 197835

**Table S3.** Hydrogen bond occupancy for the 300 ns simulation for the selected compounds and control

| Residues |  | H-Bond % occupancy | Residues |  | H-Bond % occupancy |
| --- | --- | --- | --- | --- | --- |
|  | **Control** |  |  | **638024** |  |
| ASP230 | LIG | 0.02 | LEU224 | LIG | 0.007 |
| SER233 | LIG | 20.919 | SER238 | LIG | 0.353 |
| HIS234 | LIG | 0.023 | SER238 | LIG | 0.007 |
| THR236 | LIG | 0.457 | ALA253 | LIG | 0.023 |
| ALA253 | LIG | 0.03 | TYR257 | LIG | 0.813 |
| SER305 | LIG | 0.05 | TYR257 | LIG | 0.027 |
| ILE306 | LIG | 2.403 |  | **197835** |  |
| ILE306 | LIG | 33.586 | **Residues** |  | **H-Bond % occupancy** |
| ILE306 | LIG | 0.06 | PHE226 | LIG | 0.063 |
| ILE306 | LIG | 2.257 | ALA229 | LIG | 0.09 |
| LEU307 | LIG | 0.153 | ALA229 | LIG | 0.003 |
| LEU307 | LIG | 0.047 | ALA229 | LIG | 0.013 |
| LEU307 | LIG | 0.003 | ASP230 | LIG | 0.003 |
| THR307 | LIG | 3.143 | ASP230 | LIG | 2.37 |
| THR307 | LIG | 27.406 | ASP230 | LIG | 6.06 |
| THR307 | LIG | 0.077 | ASP230 | LIG | 50.018 |
| LIG | TRP241 | 0.003 | ASP230 | LIG | 0.26 |
| LIG | GLY306 | 0.017 | HIS234 | LIG | 0.003 |
| LIG | TRP295 | 1.013 | HIS234 | LIG | 0.013 |
| LIG | ARG297 | 2.547 | THR236 | LIG | 0.097 |
| LIG | ILE298 | 3.02 | THR236 | LIG | 5.35 |
| LIG | ILE298 | 6.64 | TYR257 | LIG | 0.117 |
|  | **122623** |  | TYR257 | LIG | 0.03 |
| Residues |  | **H-Bond % occupancy** | ALA293 | LIG | 0.03 |
| PHE185 | LIG | 0.007 |  |  |  |
| HIS234 | LIG | 0.02 |  |  |  |
| ALA253 | LIG | 3.703 |  |  |  |
| MET305 | LIG | 10.693 |  |  |  |
| ILE306 | LIG | 0.15 |  |  |  |
| LEU307 | LIG | 8.186 |  |  |  |


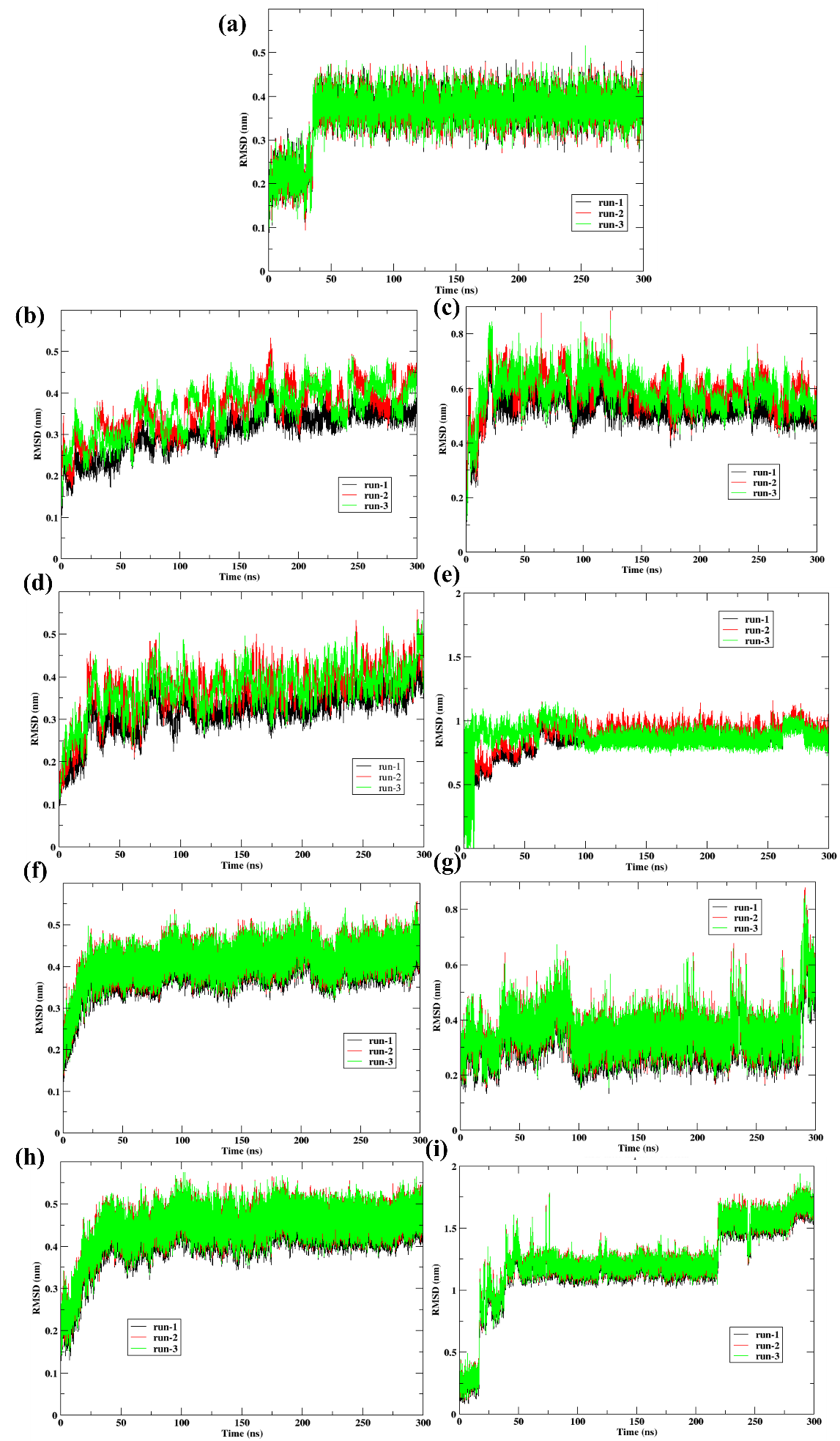


**Figure S4.** Triplicates of the 300 ns simulation RMSD of the protein of (a) Apo Protein (b) Control (d) 638024 (f) 122623 (h) 197835 and RMSD of ligand of (c) Control (e) 638024 (g) 122623 (h) 197835
